# Cardiometabolic disease costs associated with suboptimal diet in the United States: A cost analysis based on a microsimulation model

**DOI:** 10.1371/journal.pmed.1002981

**Published:** 2019-12-17

**Authors:** Thiago Veiga Jardim, Dariush Mozaffarian, Shafika Abrahams-Gessel, Stephen Sy, Yujin Lee, Junxiu Liu, Yue Huang, Colin Rehm, Parke Wilde, Renata Micha, Thomas A. Gaziano

**Affiliations:** 1 Department of Cardiovascular Medicine, Brigham and Women’s Hospital, Boston, Massachusetts, United States of America; 2 Center for Health Decision Science, Harvard T.H. Chan School of Public Health, Boston, Massachusetts, United States of America; 3 Friedman School of Nutrition Science and Policy, Tufts University, Boston, Massachusetts, United States of America; 4 Office of Community and Population Health, Montefiore Medical Center, Bronx, New York, United States of America; Harvard Medical School, UNITED STATES

## Abstract

**Background:**

Poor diet is a leading risk factor for cardiometabolic disease (CMD) in the United States, but its economic costs are unknown. We sought to estimate the cost associated with suboptimal diet in the US.

**Methods and findings:**

A validated microsimulation model (Cardiovascular Disease Policy Model for Risk, Events, Detection, Interventions, Costs, and Trends [CVD PREDICT]) was used to estimate annual cardiovascular disease (fatal and nonfatal myocardial infarction, angina, and stroke) and type 2 diabetes costs associated with suboptimal intake of 10 food groups (fruits, vegetables, nuts/seeds, whole grains, unprocessed red meats, processed meats, sugar-sweetened beverages, polyunsaturated fats, seafood omega-3 fats, sodium). A representative US population sample of individuals aged 35–85 years was created using weighted sampling from National Health And Nutrition Examination Surveys (NHANES) 2009–2012 cycles. Estimates were stratified by cost type (acute, chronic, drug), sex, age, race, education, BMI, and health insurance. Annual diet-related CMD costs were $301/person (95% CI $287–$316). This translates to $50.4 billion in CMD costs (18.2% of total) for the whole population, of which 84.3% are attributed to acute care ($42.6 billion). The largest annual per capita costs are attributed to low consumption of nuts/seeds ($81; 95% CI $74–$86) and seafood omega-3 fats ($76; 95% CI $70–$83), and the lowest are attributed to high consumption of red meat ($3; 95% CI $2.8–$3.5) and polyunsaturated fats ($20; 95% CI $19–$22). Individual costs are highest for men ($380), those aged ≥65 years ($408), blacks ($320), the less educated ($392), and those with Medicare ($481) or dual-eligible ($536) insurance coverage. A limitation of our study is that dietary intake data were assessed from 24-hour dietary recall, which may not fully capture a diet over a person's life span and is subject to measurement errors.

**Conclusions:**

Suboptimal diet of 10 dietary factors accounts for 18.2% of all ischemic heart disease, stroke, and type 2 diabetes costs in the US, highlighting that timely implementation of diet policies could address these health and economic burdens.

## Abstract 2

In a microsimulation model-based cost analysis, Gaziano and colleagues investigate the estimated cardiometabolic disease costs in the United States associated with suboptimal diet.

## Author summary

**Why was this study done?:**

- Unhealthy diet is responsible for up to 45% of all cardiometabolic disease (CMD) deaths.
- In addition to the health impact, CMDs pose a substantial economic burden.
- Despite the clear association between poor diet and CMD, the costs of a suboptimal diet pattern in the US have not been estimated.
- We sought to estimate the CMD-related cost associated with suboptimal diet in the US.

**What did the researchers do and find?:**

- We used a microsimulation model to estimate annual cardiovascular disease (CVD) and type 2 diabetes costs associated with suboptimal intake of 10 food groups. A representative US population sample of individuals aged 35–85 years was created from National Health And Nutrition Examination Surveys (NHANES) 2009–2012 cycles.
- Annual diet-related CMD costs were $301/person, which translates to $50.4 billion for the whole population (18.2% of total costs). The largest annual per capita costs are attributed to low consumption of nuts/seeds ($81) and seafood omega-3 fats ($76), and the lowest are attributed to high consumption of red meat ($3) and polyunsaturated fats ($20).
- Individual costs are highest for men ($380), those aged ≥65 years ($408), blacks ($320), the less educated ($392), and those with Medicare ($481) or dual-eligible ($536) insurance coverage.

**What do these findings mean?:**

- The CMD costs estimated to be associated with suboptimal diet highlight the need to implement diet policies to address health and economic burdens.
- The high CMD-related costs associated with suboptimal diet combined with the finding that the cost burden often falls upon the government illustrates the need for community and policy strategies to reduce this financial burden.

## Introduction

Suboptimal diet is one of the leading risk factors for poor health, both globally [1] and in the US, and is responsible for up to 45% of all cardiometabolic disease (CMD) deaths [2, 3]. Most of these CMD deaths are due to coronary heart disease (CHD), stroke, and type 2 diabetes mellitus, which in addition to the health impact, poses a substantial economic burden [4]. Considering these health and economic impacts, diet-related illnesses are among the leading priorities of our time [4]. Despite the clear association between poor diet and CMD and the overall economic burden of such conditions, the costs of a suboptimal diet pattern have only been assessed locally [5, 6] and/or focused on specific dietary factors such as sodium, saturated fats, and sugar-sweetened beverages (SSBs) [5, 7–9].

An economic analysis of the US adult population over a broad array of foods/nutrients, as well as evaluation of population subgroups’ differences, is needed. Such information would strongly reinforce, from an economic perspective, priorities in public health planning and incentives for pursuing specific strategies to change unhealthy dietary habits and ultimately reduce the cardiometabolic health–related costs. To address these gaps in knowledge, we used a microsimulation model—the Cardiovascular Disease Policy Model for Risk, Events, Detection, Interventions, Costs, and Trends (CVD PREDICT) [10]—to estimate the annual cardiometabolic costs related to suboptimal intakes of 10 dietary factors (fruits, vegetables, nuts/seeds, whole grains, unprocessed red meats, processed meats, SSBs, polyunsaturated fats, seafood omega-3 fats, and sodium), individually and jointly, among US adults; to assess diet-associated costs by CMD cost type (acute, chronic and drug-related); and to evaluate differences by key population subgroups (age, sex, race, education, and insurance status). This investigation was performed as part of the Food Policy Review and Intervention Cost-Effectiveness (Food-PRICE) Project, which is a National Institutes of Health (NIH)-funded group of collaborative researchers across the US and Europe working to identify strategies to improve the health of the US population through improved diets [11].

## Methods

### Study overview

A validated microsimulation model, the CVD PREDICT model (S1 Fig) [10], was used to estimate annual CMD costs related to suboptimal intakes of 10 dietary factors, both individually and in combination, in the US. The model incorporated data on population demographics and dietary habits from the National Health and Nutrition Examination Survey (NHANES) and age-adjusted estimates of the effect sizes for the association of 10 foods and nutrients with CHD, stroke, or type 2 diabetes as previously quantified in meta-analyses of prospective and randomized clinical trials [12], optimal population intakes of these dietary factors as previously defined [3, 12], and validated healthcare costs (S1 Table). Results were inflated to 2018 US dollars [13] and the main ones given on a 1-year per capita basis. Cost estimates focused on both prevention of onset and treatment of established CMD and were stratified by medical cost type, sex, age, race/ethnicity, education, BMI, and health insurance. Since this is not a cost-effective analysis, implementation costs and other possible financial implications of a population-based healthy diet were not included in the model. This modeling investigation was deemed exempt from human subjects’ review by the Partners Human Research Committee (Boston, MA, USA) because it used publicly available, deidentified data. We report our results as recommended for economic evaluations of health interventions using the CHEERS [14] checklist (S1 Text), and the study was based on a prespecified analysis plan (S2 Text).

### Microsimulation model

The CVD PREDICT model is a microsimulation model used to model the natural history of cardiovascular disease (CVD) through updates of individual-level CVD disease history. CVD risk factors required to run the model are sex, age, systolic blood pressure, total cholesterol, HDL cholesterol, current smoking status, and diabetes status. The model updated cardiovascular risk factors (age, total and HDL cholesterol, systolic blood pressure, diabetes status) using previously developed regressions based on NHANES data. These risk factors were used to estimate the annual risks of CVD events (CHD [cardiac arrest, myocardial infarction, angina] and stroke), and these events have acute (related to the acute hospitalization event) and postacute (i.e., not related to the acute event) effects on mortality, morbidity, and healthcare costs. The model considers a patient’s prior history of having a CVD event and populates these individuals in their respective CVD health states at the start of a model run. A comprehensive description of the model structure, input parameters, key data sources, methods for validating model performance in terms of predicting CVD, and all-cause mortality has been published elsewhere in greater detail [10].

### Population

The model was populated by weighted sampling (with replacement) of individuals aged 35–85 years from the 2009–2010 and 2011–2012 NHANES in order to create a US representative model population of 1,000,000 individuals. All individuals were simulated in yearly cycles. The baseline cohort was then expanded by adding additional groups of 35-year-olds in subsequent years, along with a conservative estimate of population growth, to produce cross-sectional outcomes at 5 years that were used to calculate the 1-year results. The model is run for 5 years to account for the fact that events assumed to happen midway in a cycle underestimate events in the first year. Half-cycle corrections at the beginning and end allow for more accurate annualized costs. The baseline characteristics of the 35-year-olds added to the cohort are given in S2 Table. All population data were obtained from publicly available datasets provided online by the National Center for Health Statistics (https://wwwn.cdc.gov/nchs/nhanes/Default.aspx).

### Dietary intakes

In addition to CVD risk-factor data, dietary intakes were estimated using nationally representative data from multiple NHANES cycles, accounting for complex survey design and sampling weights [3], to be representative of the US population aged 35–85 years. Intakes were assessed from up to two standardized 24-hour dietary recalls per person, accounting for within-person variation [3]. The optimal consumption of each food/nutrient was determined based on observed levels at which the lowest disease risk occurs [3], with further considerations of feasibility and consistency with major guidelines [15]. Optimal consumption could be zero for unhealthy foods or some positive value for a healthy nutrient. Optimal metrics and units for each dietary factor were defined to be consistent with definitions used in epidemiological studies that provided evidence on diet–disease relative risks [16]. The methods, criteria, and results for review, identification, and assessment of dietary factors for inclusion in etiologic diet–disease analyses have been described [3]. Briefly, our primary determinations were based on Bradford‐Hill criteria, including evidence on strength/consistency, temporality, coherence, specificity, analogy, plausibility, biological gradient, and supportive experimental data, reviewed and graded independently and in duplicate [12]. We included factors that were considered to have at least probable or convincing evidence for causal relationships. For each identified diet–disease relationship, we relied on previously published multiple systematic searches of PubMed that identified meta-analyses of randomized controlled trials or prospective cohort studies evaluating these specific dietary factors and CMD; or we performed de novo meta-analyses according to the PRISMA guidelines [12]. Detailed information on the 10 food/nutrients used in the analysis is provided in S3 Text.

Among the factors with at least probable evidence for causal diet–CMD relationships [12], two (dietary potassium, yogurt) were not included for consistency with our recent publication on US diet–CMD burdens [3]; two others (trans fat, glycemic load) were not included because of insufficient information in NHANES on their dietary intakes; and others (fish, fiber, potassium) were not included because of major overlap with other dietary factors already included in the present analysis. Several other potentially relevant dietary factors were evaluated and not included because of still-emerging and not-yet-probable or convincing evidence on causal relationships, including, for example, total monounsaturated fats, vitamin D, magnesium, calcium, antioxidant vitamins, milk, cheese, cocoa, coffee, tea, and varying definitions of processed foods. Because several of these factors may turn out to have important causal effects, our findings should serve as a foundation for future analyses as further evidence on these relationships accumulates. Thus, the present results should be considered a conservative estimate of the diet-attributable costs of CMDs, which are potentially higher.

### Analyses stratification

The estimates were stratified by sex (male and female), age (35–49, 50–64, and ≥65 years), race/ethnicity (white: non‐Hispanic white; black: non‐Hispanic black; Hispanic: Mexican American/other Hispanic; and other: other race/mixed race), education (<high school: less than a high school degree; high school: high school degree/equivalent or some college; and college: ≥4‐year college degree), and health insurance categories (private, Medicare, Medicaid, dual eligible, other government, and no coverage). In addition, we stratified by BMI. A detailed description of health insurance stratification is provided in S4 Text. Baseline characteristics of the modeled population stratified by health insurance are described in S3 Table and S4 Table.

In addition to health insurance stratification, we also assessed the healthcare costs proportionally to their ultimate cost-bearers. The ultimate cost-bearers for healthcare are (1) households (who pay out-of-pocket costs and some insurance premiums), (2) the government (which funds public insurance programs), and (3) third parties (employers that subsidize insurance premiums and workers’ compensation benefits, hospitals that absorb unreimbursed medical spending, charitable entities, and others). For each consumer category, we determined the proportion of expenditures for households, government, and third parties by using data from MEPS [17] and the Centers for Medicare and Medicaid Services (CMS) [18]. Full methods and sources are presented in S5 Text, S5 Table, S6 Text, S6 Table and S7 Table.

Detailed health outcomes stratified by food groups, sex, age group, race, education, and health insurance are provided in S8 Table, S9 Table, S10 Table and S11 Table, and 5-year dietary-related costs of CMD are listed in S12 Table and S13 Table.

### Costs

An analysis of healthcare costs based on the NHANES survey information on consumption of the 10 food/nutrients was generated by the model and compared to a hypothetical situation in which the consumptions of the 10 food/nutrients were optimal. Each dietary factor was evaluated individually and in combination. Costs include acute costs (related to the acute hospitalization event), annual chronic costs of CVD and diabetes (not related to the acute event or drugs), and drug-related costs. The discount rate used for all costs is 3% in accordance with the Second Panel on Cost-Effectiveness in Health and Medicine [19]. Detailed information on cost estimates is presented in the supporting information (S1 Table).

Descriptive statistics for the model population were generated using the statistical software packages Stata 14 (College Station, TX, USA) and SAS 9.4 (Cary, NC, USA).

## Results

The mean age of the modeled cohort was 54.9 (±12.7) years, with females (52.7%) and whites (72.2%) representing the majority of the cohort. The percentage of individuals with a history of angina, myocardial infarction, and stroke was 2.6%, 4.3%, and 3.3%, respectively. Baseline characteristics of the cohort, optimal consumption of the 10 food/nutrients assessed, mean population consumption, and percentage of individuals with better than optimal or optimal consumption are given in Table 1. The highest percentage of individuals with optimal consumption were found for SSBs (48.1%), red meat (35.7%), and processed meat (32.1%), whereas the lowest were for whole grains (0.7%), sodium (2.4%), and vegetables (7.9%).

**Table 1:** Modeled population description.

Variables	Mean or Proportion	Standard Deviation	
Age (years)	54.9	12.7	
35–49 years (%)	38.7		
50–64 years (%)	37.8		
≥65 years (%)	23.5		
Sex			
Female (%)	52.7		
Race*			
White (%)	72.2		
Black (%)	10.1		
Hispanic (%)	11.7		
Others (%)	6.0		
Education^†^			
<High school (%)	17.5		
High school (%)	51.1		
College (%)	31.4		
Health insurance^‡^			
Private (%)	55.7		
Medicare (%)	19.5		
Medicaid (%)	3.3		
Dual eligible (%)	1.3		
Other government (%)	5.3		
No coverage (%)	14.9		
Body mass index (kg/m^2^)	29.2	6.6	
Systolic blood pressure (mmHg)	123.8	17.5	
Diastolic blood pressure (mmHg)	72.1	11.6	
Total cholesterol (mg/dL)	202.3	42.5	
HDL cholesterol (mg/dL)	54.1	16.8	
LDL cholesterol (mg/dL)	118.5	36.6	
Triglycerides (mg/dL)	134.7	108.0	
History of diabetes (%)	11.4		
Current smoker (%)	16.6		
Current hypertension treatment (%)	35.1		
Angina (%)	2.6		
Myocardial infarction (%)	4.3		
Stroke (%)	3.3		
	Mean Consumption	Standard Deviation	Optimal^§^ (%)
Fruits excluding fruit juices, g/day	120.6	145.5	9.2
Vegetables including legumes, g/day	188.2	153.4	7.9
Nuts/seeds, g/day	12.5	29.0	19.5
Whole grains, g/day	21.9	26.1	0.7
Red meats, unprocessed, g/day	46.9	51.0	35.7
Processed meats, g/day	30.7	38.3	32.1
SSBs, 8-oz (236.5-ml) servings/day	1.0	1.5	48.1
PUFAs, % energy replacing carbohydrates or saturated fats	7.8	2.7	11.4
Seafood omega-3 fats, mg/day	98.1	187.8	9.1
Sodium, mg/day	3,481.5	965.7	2.4

*Race—white: non‐Hispanic white; black: non‐Hispanic black; Hispanic: Mexican American/other Hispanic; and other: other race/mixed race.

^†^Education—<high school: less than high school degree; high school: high school degree/equivalent or some college; college: ≥4‐year college degree.

^‡^Health insurance—Private includes private, single service plan, private plus other government, other coverage; Medicare includes Medicare, Medigap, Medicare plus other government, Medicare plus private; Medicaid includes only Medicaid; dual eligible includes Medicare plus Medicaid; and other government includes other government, state-sponsored, military.

^§^Percentage of individuals with optimal or better than optimal consumption of the dietary item (fruits excluding fruit juices: 300 g/day; vegetables including legumes: 400 g/day; nuts/seeds: 20.2 g/day (5 1-oz servings/week); whole grains: 125 g/day (2.5 50-g servings/day); red meats, unprocessed: 14.3 g/day (1 100-g serving/week); processed meats: no intake; SSBs: no intake; PUFAs: 11% energy replacing carbohydrates or saturated fats; seafood omega-3 fats: 250 mg/day; sodium: 2,000 mg/day).

Abbreviations: HDL, high-density lipoprotein; LDL, low-density lipoprotein; PUFA, polyunsaturated fatty acid; SSB, sugar-sweetened beverage

Acute, chronic, drug-related, and total costs of CMDs related to a suboptimal diet and stratified by the 10 food/nutrient groups were estimated in a 1-year period. Those results are presented in US dollars per person in Table 2. The 1-year total cost of an unhealthy diet was $301/person/year (acute $254, chronic $43, and drug $4). Diabetes accounted for approximately 50% ($21) of the annual chronic costs, and the rest of the chronic costs were attributable to CVD. Individually, the food/nutrients consumed below optimal levels that imposed the highest CMD economic burden were nuts/seeds ($81), seafood omega-3 fats ($76), and processed meats ($61). Oppositely, high consumption of red meat ($3), polyunsaturated fats ($20), and sodium ($23) was associated with the lowest cost per person.

**Table 2:** Annual cardiometabolic costs* among US adults aged ≥35 years associated with suboptimal dietary habits and stratified by sex, age, race, education, and health insurance.

Suboptimal Diet and Food/Nutrient Ggroups		Total Cost (95% CI)	Acute^†^	Chronic^‡^	Drug^§^
Suboptimal diet^||^		301 (287–316)	254	43	4
Fruits excluding fruit juices, g/day		57 (53–61)	53	4	0
Vegetables including legumes, g/day		60 (56–65)	56	4	0
Nuts/seeds, g/day		81 (74–86)	70	11	0
Whole grains, g/day		45 (42–48)	32	13	0
Red meats, unprocessed, g/day		3 (2.8–3.5)	0	3	0
Processed meats, g/day		61 (53–66)	50	11	0
Sugar-sweetened beverages, 8-oz (236.5-ml) servings/day		58 (54–61)	47	11	0
PUFAs, % energy replacing carbohydrates or saturated fats		20 (19–22)	19	1	0
Seafood omega-3 fats, mg/day		76 (70–83)	71	5	0
Sodium, mg/day		23 (22–25)	18	2	3
Suboptimal diet^||^ stratifications					
Sex	Male	380	323	52	5
	Female	221	184	33	3
Age	35–49 years	144	105	36	5
	50–64 years	325	278	42	5
	65–84 years	408	366	37	5
Race**	White	299	252	43	4
	Black	320	270	43	6
	Hispanic	276	229	42	4
	Others	282	237	42	3
BMI	<30	261	215	41	4
	≥30	361	310	45	5
Education^††^	<High school	392	340	46	5
	High school	326	274	46	5
	College	208	168	36	3
Health insurance^‡‡^	Private	231	186	41	4
	Medicare	481	431	45	5
	Medicaid	318	270	42	5
	Dual eligible	536	485	45	5
	Other government	300	253	43	4
	No coverage	316	264	48	4

*Values given in 2018 US dollars.

^†^Acute costs: related to the acute hospitalization event.

^‡^Chronic costs: not related to the acute event or drugs.

^§^Drug costs: drug-related costs.

^||^Suboptimal diet: combination of 10 dietary factors.

**Race—white: non‐Hispanic white; black: non‐Hispanic black; Hispanic: Mexican American/other Hispanic; and other: other race/mixed race.

^††^Education—<high school: less than high school degree; high school: high school degree/equivalent or some college; college: ≥4‐year college degree.

^‡‡^Health insurance—Private includes private, single service plan, private plus other government, other coverage; Medicare includes Medicare, Medigap, Medicare plus other government, Medicare plus private; Medicaid includes only Medicaid; dual eligible includes Medicare plus Medicaid; and other government includes other government, state-sponsored, military.

Abbreviation: PUFA, polyunsaturated fatty acid

Annual costs of CMD attributed to unhealthy diet by sex, age, race, education level, and health insurance are also presented in Table 2. In these stratifications, costs were higher in men ($380) versus women ($221), at older (≥65 years) ($408) versus younger (<65 years) ($251) ages, in blacks ($320) and whites ($299) versus Hispanics ($276), among those with lower ($392) versus higher ($208) education, and in Medicaid ($318), Medicare ($481), and especially dual-eligible individuals ($536) versus private insurance ($231).

The contribution of each food group to the total cost of CMD is shown in Table 3. Nuts/seeds had the strongest impact in the overall cost of CMD (17%), followed by seafood omega-3 (16%) and processed meat (13%). Red meat (1%), polyunsaturated fatty acids (4%), and sodium (5%) were the dietary factors with the weakest impact. When the same analysis was conducted stratified by sex, age, race, education, and health insurance, the results were mostly homogeneous across the groups. (Table 3)

**Table 3:** Dietary factors’ proportion of cardiometabolic costs by sex, age, race, education, and health insurance.

		Fruits (%)	Vegs (%)	Nuts (%)	Grains (%)	Red Meat (%)	Processed Meat (%)	SSB (%)	PUFA (%)	Omega-3 (%)	Sodium (%)
All*		11.8	12.4	16.6	9.3	0.7	12.6	12.0	4.2	15.7	4.7
Sex	Female	12.2	13.4	16.8	10.3	0.6	10.3	10.8	3.8	15.4	6.5
	Male	11.6	12.2	16.5	8.8	0.5	14.1	12.1	4.4	15.8	3.9
Age group	≥65 years	12.8	13.5	17.2	9.0	0.2	12.1	7.5	4.1	16.4	7.3
	<65 years	11.1	11.7	16.7	9.3	0.8	13.5	13.6	4.1	15.6	3.6
Race^†^	Hispanic	11.2	10.7	19.5	9.6	0.7	9.8	13.7	4.7	16.0	4.1
	Black	11.6	12.6	16.4	8.7	0.5	13.5	15.0	3.4	13.4	4.9
	White	12.0	12.8	15.9	9.5	0.7	13.1	11.0	4.3	15.9	4.9
Health insurance^‡^	Private	11.6	12.1	15.7	9.9	0.7	14.2	11.6	4.1	15.5	4.6
	Medicare	12.7	13.8	17.3	9.0	0.3	11.4	8.6	3.9	16.3	6.8
	Medicaid	11.7	12.9	17.6	9.0	0.6	12.4	13.1	4.2	14.8	3.8
	Dual eligible	12.2	13.5	17.8	9.0	0.3	11.1	14.8	4.3	13.3	3.7
	Other government	11.3	13.4	16.4	9.0	0.7	12.4	11.9	4.7	16.0	4.2
	No coverage	10.7	10.7	18.2	8.5	0.5	12.3	16.2	4.4	15.4	3.0

*The proportion of costs attributed to a nonideal dietary consumption of the specific food or nutrient. Top row shows impact of food group for all individuals, and remaining rows show impacts stratified by sex, age, race, and type of insurance.

^†^Race—white: non‐Hispanic white; black: non‐Hispanic black; Hispanic: Mexican American/other Hispanic; and other: other race/mixed race.

^‡^Health insurance—Private includes private, single service plan, private plus other government, other coverage; Medicare includes Medicare, Medigap, Medicare plus other government, Medicare plus private; Medicaid includes only Medicaid; dual eligible includes Medicare plus Medicaid; and other government includes other government, state-sponsored, military.

Abbreviations: PUFA, polyunsaturated fatty acid; SSB, sugar-sweetened beverage

The estimated annual dietary-related cost of CMD in the US is $50.4 billion (S2 Fig). Breaking down the costs in acute, chronic, and drug-related costs, acute costs represent 84.3% of this total ($42.6 billion). Regarding chronic costs, from the $7.2 billion spent, $3.6 billion was related to CVD, whereas the other $3.6 billion was spent in diabetes care. The overall costs stratified by food group are provided in Table 4.

**Table 4:** National annual costs associated with nonoptimal daily consumption of individual dietary factors.

Food Category	Ideal Daily Consumption	Annual Cardiometabolic Costs* (95% CI)
Fruits	300 g/day	9.552 (8.904–10.248)
Vegetables	400 g/day	10.055 (9.408–10.92)
Nuts/seeds	20 g/day	13.574 (12.432–14.448)
Whole grains	125 g/day	7.541 (7.056–8.064)
Red meat	14.3 g/day	0.503 (0.4704–0.588)
Sugar-sweetened beverages	0 oz/day	10.223 (8.904–11.088)
Processed meat	0 g/day	9.72 (9.072–10.248)
Polyunsaturated fatty acids	11% Energy/day	3.352 (3.192–3.696)
Seafood omega-3	250 mg/day	12.736 (11.76–13.944)
Sodium	2000 mg/day	3.854 (3.696–4.2)

*Values given in 2018 US billions of dollars. Total cost does not reflect sum of individual components based on the assumption that the benefits of the 10 food groups are not independent.

The annual dietary-related costs of CMD by insurance categories and ultimate cost-bearer (US$) by insurance type are presented for the US population in S3 Fig. For those with a private insurer, households could see returns in lower future premiums or out-of-pocket expenses from an optimal diet. However, in every case, the government or third-party payer is paying for the majority of these costs. Further, with the exceptions of the private and no coverage categories, for the four other insurance categories, the government is responsible for the majority of these diet-related costs (Medicare = 70.3%, Medicaid = 95.2%, dual eligible = 93.2%, and other government = 59.1%).

## Discussion

Using a microsimulation model, we estimated the annual CMD costs related to the suboptimal intake of 10 common dietary factors associated with CVD benefit or harm to be $50.4 billion. Considering that the annual direct cost of CVD and diabetes in the US was estimated at $276.3 billion [20], our estimates suggest that 18.2% of this cost is attributable to an unhealthy diet pattern. Annual diet-related CMD costs per capita were $301/person, and the costs are attributed to low consumption of nuts/seeds ($81) and seafood omega-3 fats ($76), and the lowest were attributed to high consumption of red meat ($3) and polyunsaturated fats ($20). Several studies have evaluated whether healthier foods or diets cost more [21–25], and many others, which focused on disease related costs, were restricted to specific dietary factors [5, 6] and/or did not assess nationwide costs [5, 7–9]. This study presents both an overall US population analysis and a view of the impact of the major nutritional contributors simultaneously on CMD costs.

Costs attributed to a particular dietary group are driven by two factors: the relative-risk reduction per unit change in consumption and how far an individual is from the ideal consumption of the food item. Cost-savings could come from a large change in consumption of a healthy item even if the reduction in disease is moderate for that dietary factor or from a minor change in consumption of dietary factor associated with a large relative-risk reduction in CVD. Individuals who are consuming at or near the ideal amounts of the dietary factors cannot improve their risk or savings much, and the converse is true for those consuming far from the ideal levels. For example, higher diet-related costs of CMD were associated with suboptimal consumption of foods/nutrients that are not usually thought of as having a great impact on healthcare costs, such as nuts/seeds and seafood omega-3 [26]. This was because the mean consumption of these dietary factors in the US is extremely low, and thus, there is much to gain from an increase to ideal levels of consumption. Further, although changes in SSB consumption can result in a statistically significant relative-risk reduction in stroke, diabetes, and CHD [3] (all diseases that are included in our model), 48.1% of the modeled US population was already at the optimal level of SSB consumption (no SSB) and, therefore, cannot improve their CMD risk. These results suggest that from an economic perspective, intervention policies not just focused on the benefit or harm of dietary factors but also focused on how far the population is from achieving ideal consumption are more likely to have economic benefit.

Although nutrition interventions constitute a highly efficient component of a strategy to reduce the growing CVD-related costs linked to poor nutrition [27], extensive analyses of these costs were missing in the literature. We conducted a real-world assessment, including analyses stratified by the actual payers (health insurance categories). The stratification of the results by health insurance groups highlights the need for payers to get more involved in the efforts of optimizing the lifestyle patterns of the US population. Interventions to incentivize healthier dietary behavior driven by the insurance companies can be implemented in order to improve diet habits in the US population [28]. Well-known and generally well-accepted examples are the high-deductible health plans in which members are more likely to adopt healthy behaviors to reduce future costs of chronic diseases [29]. Since food choice is complicated and integral to the human experience, it may be a reasonable strategy for insurers to subsidize healthy foods instead of dictate the diet to reduce diet-related costs [28].

A potential limitation of this analysis is the expected underestimated costs of the unhealthy diet. This underestimation is based on the assumption that the health benefits (and subsequently discounted costs) only occur in those with suboptimal diet consumption. Since this principle was applied to all food/nutrients groups, we expect our results to be under the real CVD-related costs of an unhealthy diet. Another limitation is that we estimated the potential healthcare saving based on a hypothetical situation of optimal intakes and did not assess the feasibility of achieving intakes. Additionally, dietary intake data assessed from 24-hour dietary recalls per person may be limited to characterize diet over a person's life span and are subject to measurement errors, such as underestimating portion sizes or failing to include foods and beverages that are either forgotten or consumed infrequently [30, 31]. Although potential errors were further reduced by energy adjustment and distributions were corrected for within-person variation [3], the dietary consumption information is probably underestimated. We were also not able to stratify by all factors that might affect cost and CVD risk such as physical activity. However, stratification by age, BMI, and educational level revealed that those risk-factor levels associated with higher CVD risk also were likely to have greater cost-savings if ideal diets could be achieved. Thus, efforts to improve diets among the elderly, those with higher BMI, and those with lower educational attainment could yield greater savings. A limitation of the model itself is that it does not include all possible health costs that may be attributed to improved diet such as reductions in hypertensive heart failure, atrial fibrillation, or renal failure attributed to improved blood pressure. Finally, we do not include all potential dietary factors that might affect cardiometabolic conditions.

The purpose of this study was not to conduct a cost-effectiveness analysis of a dietary intervention but rather to provide economic data related to a previous publication from our group in which the association between the 10 dietary factors and mortality from heart disease, stroke, and type 2 diabetes was assessed [3]. Although the CVD PREDICT model allows cost-effectiveness assessments, there are no available data on costs of interventions aiming to achieve an optimal diet pattern for all 10 dietary factors from our analyses. Individual policy actions to improve the optimal consumption of one or more of these dietary factors are being assessed as the data for both the cost and the effectiveness of such interventions are explored, such as for changes to reduce SSB consumption [32] via taxes and increase fruit and vegetable consumption through rebates in the Supplemental Nutrition Assistance Program (SNAP) [33].

In conclusion, the 1-year estimated CMD cost per capita associated with an unhealthy diet in the US among those aged 35–85 years is $301, translating to a population total cost of $50.4 billion. Considering the high annual costs associated with CMD in the US that we estimate to be attributable to suboptimal diet, and the participation of the government as the majority payer of such costs, these findings should motivate the healthcare and policymaking communities to implement strategies to reduce this financial and health burden.

## Supporting information

S1 Text CHEERS checklist. CHEERS, Consolidated health economic evaluation reporting standards.
(DOCX)

S2 Text Prospective analytic plan. June 8, 2017.
(DOCX)

S3 Text Detailed information on the 10 foods/nutrients assessed. (DOCX)

S4 Text Description of health insurance stratification. (DOCX)

S5 Text Attributing proportion of healthcare costs by consumer category and ultimate cost-bearer. (DOCX)

S6 Text Modeled costs estimates. (DOCX)

S1 Fig The CVD PREDICT microsimulation model. CVD PREDICT, Cardiovascular Disease Policy Model for Risk, Events, Detection, Interventions, Costs, and Trends.
(DOCX)

S2 Fig Pathways for cardiometabolic disease prevention associated with optimal dietary habits and annual cardiometabolic costs. (DOCX)

S3 Fig Annual ultimate cost-bearer of cardiometabolic costs among US adults aged ≥35 years associated with suboptimal dietary habits, by health insurance type. (DOCX)

S1 Table Costs used in the CVD PREDICT model parameter. CVD PREDICT, Cardiovascular Disease Policy Model for Risk, Events, Detection, Interventions, Costs, and Trends.
(DOCX)

S2 Table Model population description for adults aged 35 years. (DOCX)

S3 Table Modeled population description by health insurance. (DOCX)

S4 Table Modeled population dietary intake by health insurance. (DOCX)

S5 Table Distribution of healthcare cost by consumer category and ultimate cost-bearer. (DOCX)

S6 Table 2014 personal healthcare expenditure, by sources of payments and ultimate cost-bearer. (DOCX)

S7 Table Dietary factors associated with cardiometabolic outcomes and standardized magnitudes of effect sizes. (DOCX)

S8 Table Five-year health outcomes (per million) by food/nutrient group. (DOCX)

S9 Table Five-year health outcomes (per million) by sex, age, race, and health insurance. (DOCX)

S10 Table One-year health outcomes (per million) by food/nutrient group. (DOCX)

S11 Table One-year health outcomes (per million) by sex, age, race, and health insurance. (DOCX)

S12 Table Five-year dietary-related costs of cardiometabolic disease per adult (US$) by food/nutrient group. (DOCX)

S13 Table Five-year dietary-related costs of cardiometabolic disease per adult (US$) by sex, age, race, and health insurance. (DOCX)

## Abbreviations

CHD: coronary heart disease
CMD: cardiometabolic disease
CMS: Centers for Medicare and Medicaid Services
CVD PREDICT: Cardiovascular Disease Policy Model for Risk, Events, Detection, Interventions, Costs, and Trends
CVD: cardiovascular disease
Food-PRICE: Food Policy Review and Intervention Cost-Effectiveness
NHANES: National Health And Nutrition Examination Surveys
NIH: National Institutes of Health
SNAP: Supplemental Nutrition Assistance Program
SSB: sugar-sweetened beverage
