# Supplementary material for: Cardiometabolic disease costs associated with suboptimal diet in the United States: A cost analysis based on a microsimulation model
Source: PLoS Med. 2019 Dec 17;16(12):e1002981. doi: 10.1371/journal.pmed.1002981 (PMC6917211; doi:10.1371/journal.pmed.1002981)
Supplement: S2 Text — June 8, 2017. (DOCX) [file pmed.1002981.s002.docx]

**S2.Text. Prospective Analytic Plan. June 8, 2017**

**TITLE (initial):** Dietary costs of cardiovascular disease in the United States – estimates from a Cardiovascular Disease Policy Micro-Simulation Model

**AUTHORS** : Thiago Veiga Jardima, Shafika Abrahams-Gessel, Stephen Sy, Dariush Mozaffarian, Renata Micha , Thomas A. Gaziano (TBD).

**INTRODUCTION**

- Suboptimal diet is the leading cause of poor health, both globally(1) and in the US(2). Most of this due to cardiometabolic diseases such as coronary heart disease (CHD), stroke and type 2 diabetes mellitus, which in addition to the health impact poses a substantial economic burden (3).
- Currently, cardiovascular disease and diabetes costs in the US are estimated to approach $700 billion per year(4, 5).
- The associations of suboptimal diet with overall health at the population level have been estimated in modeling studies (1, 2, 6, 7).
- Costs of a suboptimal diet pattern have only been assessed locally(8, 9) and/or focused on specific dietary factor(8, 10-12).
- Decision-analytic models can play important roles in guiding policy on a variety of health matters. The advantage of such models lies in their ability to simulate risk health and cost outcomes that would otherwise not be observable or would take several years to develop. Short-term and long-term outcomes can be calculated, and health-improving interventions can be assessed for both comparative and cost-effectiveness(13).
- An US population estimate extensive to a higher number of food/nutrients is needed. Such information would strongly reinforce from an economic perspective the priorities in public health planning, incentive pursuing strategies to change unhealthy dietary habits and ultimately reduce the cardiometabolic health-related costs.

**AIMS**

To estimate the acute and chronic costs of cardiometabolic diseases related to suboptimal intakes of 10 dietary factors, individually and jointly, among US adults in 2012, using a microsimulation cost of events model - the Cardiovascular Disease Policy Model for Risk, Events, Detection, Interventions, Costs, and Trends (CVD PREDICT)

**METHODS**

A validated microsimulation model, the CVD PREDICT model(13), will be used to estimate the costs of cardiometabolic diseases related to suboptimal intakes of 10 dietary factors in the United States, individually and in combination. The model simulated cardiovascular disease (CVD) progression and events for individuals drawn from 2009 – 2012 National Health and Nutrition Examination Survey (NHANES). A lifetime horizon will be used in our base-case analysis. Consumption of 10 foods/nutrients associated with cardiometabolic diseases will be assessed: fruits, vegetables, nuts/seeds, whole grains, unprocessed red meats, processed meats, sugar-sweetened beverages (SSBs), polyunsaturated fats, seafood omega-3 fats, and sodium. Standardized magnitudes of effect sizes by age for each food/nutrient published by Micha et al(7) will be used to populate the model.

**REFERENCES**

**1. Lim SS, Vos T, Flaxman AD, Danaei G, Shibuya K, Adair-Rohani H, et al. A comparative risk assessment of burden of disease and injury attributable to 67 risk factors and risk factor clusters in 21 regions, 1990–2010: a systematic analysis for the Global Burden of Disease Study 2010. The Lancet. 2012;380(9859):2224-60.**

**2. Murray CJ, Atkinson C, Bhalla K, Birbeck G, Burstein R, Chou D, et al. The state of US health, 1990-2010: burden of diseases, injuries, and risk factors. Jama. 2013;310(6):591-608. Epub 2013/07/12.**

**3. Mozaffarian D. Dietary and Policy Priorities for Cardiovascular Disease, Diabetes, and Obesity. A Comprehensive Review. 2016;133(2):187-225.**

**4. Economic costs of diabetes in the U.S. in 2012. Diabetes Care. 2013;36(4):1033-46. Epub 2013/03/08.**

**5. Heidenreich PA, Trogdon JG, Khavjou OA, Butler J, Dracup K, Ezekowitz MD, et al. Forecasting the future of cardiovascular disease in the United States: a policy statement from the American Heart Association. Circulation. 2011;123(8):933-44. Epub 2011/01/26.**

**6. Forouzanfar MH, Alexander L, Anderson HR, Bachman VF, Biryukov S, Brauer M, et al. Global, regional, and national comparative risk assessment of 79 behavioural, environmental and occupational, and metabolic risks or clusters of risks in 188 countries, 1990–2013: a systematic analysis for the Global Burden of Disease Study 2013. The Lancet.386(10010):2287-323.**

**7. Micha R, Peñalvo JL, Cudhea F, Imamura F, Rehm CD, Mozaffarian D. Association Between Dietary Factors and Mortality From Heart Disease, Stroke, and Type 2 Diabetes in the United States. Jama. 2017;317(9):912-24.**

**8. Gase LN, Kuo T, Dunet D, Schmidt SM, Simon PA, Fielding JE. Estimating the Potential Health Impact and Costs of Implementing a Local Policy for Food Procurement to Reduce the Consumption of Sodium in the County of Los Angeles. American Journal of Public Health. 2011;101(8):1501-7.**

**9. Akanni O, Smith M, Ory M. Cost-Effectiveness of a Community Exercise and Nutrition Program for Older Adults: Texercise Select. International journal of environmental research and public health. 2017;14(5):545.**

**10. Palar K, Sturm R. Potential societal savings from reduced sodium consumption in the U.S. adult population. American journal of health promotion : AJHP. 2009;24(1):49-57. Epub 2009/09/16.**

**11. Doll TM, Fulgoni VL, Zhang Y, Reimers KJ, Packard PT, Astwood JD. Potential Health Benefits and Medical Cost Savings from Calorie, Sodium, and Saturated Fat Reductions in the American Diet. American Journal of Health Promotion. 2009;23(6):412-22.**

**12. Smith-Spangler CM, Juusola JL, Enns EA, Owens DK, Garber AM. Population strategies to decrease sodium intake and the burden of cardiovascular disease: a cost-effectiveness analysis. Ann Intern Med. 2010;152(8):481-7.**

**13. Pandya A, Sy S, Cho S, Alam S, Weinstein MC, Gaziano TA. Validation of a Cardiovascular Disease Policy Micro-Simulation Model using Both Survival and Receiver Operating Characteristic Curves. Medical decision making : an international journal of the Society for Medical Decision Making. 2017:272989x17706081. Epub 2017/05/12.**

**Table 1 - Descriptive statistics**

|  | Overall population | |
| --- | --- | --- |
|  | Mean or proportion | SD |
| Age (years) | 54.9 | 12.7 |
| Female (%) | 52.7 |  |
| African Americans (%) | 10.1 |  |
| BMI (kg/m2) | 29.2 | 6.6 |
| SBP (mmHg) | 123.8 | 17.5 |
| DBP (mmHg) | 72.1 | 11.6 |
| Cholesterol (mgrams/dayL) | 202.3 | 42.5 |
| HDL (mgrams/dayL) | 54.1 | 16.8 |
| LDL (mgrams/dayL) | 118.5 | 36.6 |
| Trig (mgrams/dayL) | 134.7 | 108.0 |
| History of Diabetes (%) | 11.4 |  |
| Current smoker (%) | 16.6 |  |
| Current hypertension treatment (%) | 35.1 |  |
| Angina (%) | 2.6 |  |
| Myocardial infarction (%) | 4.3 |  |
| Sroke (%) | 3.3 |  |

**Table 2 - Dietary descriptive statistics**

|  | Optimal consumption | Overall population | |
| --- | --- | --- | --- |
|  | Mean | Mean | SD |
| Fruits excluding fruit juices, grams/day | 300 | 120.6 | 145.5 |
| Vegetables including legumes, grams/day | 400 | 188.2 | 153.4 |
| Nuts/seeds, grams/day | 20.2 (5 1-oz servings/wk) | 12.5 | 29.0 |
| Whole grains, grams/day | 125 (2.5 50-g servings/d) | 21.9 | 26.1 |
| Red meats, unprocessed, grams/day | 14.3 (1 100-g serving/wk) | 46.9 | 51.0 |
| Processed meats, grams/day | No intake | 30.7 | 38.3 |
| SSBs, 8-oz servings/d | No intake | 1.0 | 1.5 |
| PUFAs,%energy replacing carbohydrates or saturated fats | 11 | 7.8 | 2.7 |
| Seafood omega-3 fats, mgrams/day | 250 | 98.1 | 187.8 |
| Sodium, mgrams/day | 2000 | 3481.5 | 965.7 |

**Relative risks (Micha, R., et al., Association Between Dietary Factors and Mortality From Heart Disease, Stroke, and Type 2 Diabetes in the United States. Jama, 2017. 317(9): p. 912-924).**

| **Fruits excluding fruit juices, grams/day** | |  |  |  |  |  |  |  |
| --- | --- | --- | --- | --- | --- | --- | --- | --- |
|  | **units of RR** | **100** | **grams/day** | **Age** | **RR CHD** | **RR Is Stroke** | **RR He Stroke** | **RR Diabetes** |
|  | **optimal** | **300** | **grams/day** | **25-34** | **0.92** | **0.83** | **0.63** |  |
|  | **mean consumption** | **120.6** | **grams/day** | **35-44** | **0.92** | **0.83** | **0.64** |  |
|  |  |  |  | **45-54** | **0.93** | **0.86** | **0.69** |  |
|  |  |  |  | **55-64** | **0.94** | **0.88** | **0.73** |  |
|  |  |  |  | **65-74** | **0.95** | **0.9** | **0.77** |  |
|  |  |  |  | **≥75** | **0.97** | **0.94** | **0.86** |  |
| **Vegetables including legumes, grams/day** | |  |  |  |  |  |  |  |
|  | **units of RR** | **100** | **grams/day** | **Age** | **RR CHD** | **RR Is Stroke** | **RR He Stroke** | **RR Diabetes** |
|  | **optimal** | **400** | **grams/day** | **25-34** | **0.93** | **0.76** | **0.76** |  |
|  | **mean consumption** | **188.2** | **grams/day** | **35-44** | **0.93** | **0.77** | **0.77** |  |
|  |  |  |  | **45-54** | **0.94** | **0.8** | **0.8** |  |
|  |  |  |  | **55-64** | **0.95** | **0.83** | **0.83** |  |
|  |  |  |  | **65-74** | **0.96** | **0.86** | **0.86** |  |
|  |  |  |  | **≥75** | **0.98** | **0.92** | **0.92** |  |
| **Nuts/seeds, grams/day** | |  |  |  |  |  |  |  |
|  | **units of RR** | **1** | **oz/week** | **Age** | **RR CHD** | **RR Is Stroke** | **RR He Stroke** | **RR Diabetes** |
|  | **optimal** | **20.2** | **grams/day** | **25-34** | **0.89** |  |  | **0.95** |
|  | **optimal** | **5** | **oz/week** | **35-44** | **0.89** |  |  | **0.95** |
|  | **mean consumption** | **12.5** | **grams/day** | **45-54** | **0.91** |  |  | **0.96** |
|  |  |  |  | **55-64** | **0.92** |  |  | **0.97** |
|  |  |  |  | **65-74** | **0.93** |  |  | **0.97** |
|  |  |  |  | **≥75** | **0.96** |  |  | **0.98** |
| **Whole grains, grams/day** | |  |  |  |  |  |  |  |
|  | **units of RR** | **50** | **grams/day** | **Age** | **RR CHD** | **RR Is Stroke** | **RR He Stroke** | **RR Diabetes** |
|  | **optimal** | **125** | **grams/day** | **25-34** | **0.95** | **0.88** | **0.88** | **0.83** |
|  | **mean consumption** | **21.9** | **grams/day** | **35-44** | **0.95** | **0.88** | **0.88** | **0.83** |
|  |  |  |  | **45-54** | **0.96** | **0.9** | **0.9** | **0.86** |
|  |  |  |  | **55-64** | **0.97** | **0.91** | **0.91** | **0.88** |
|  |  |  |  | **65-74** | **0.97** | **0.93** | **0.93** | **0.9** |
|  |  |  |  | **≥75** | **0.98** | **0.96** | **0.96** | **0.94** |
| **Red meats, unprocessed, grams/day** | |  |  |  |  |  |  |  |
|  | **units of RR** | **100** | **grams/day** | **Age** | **RR CHD** | **RR Is Stroke** | **RR He Stroke** | **RR Diabetes** |
|  | **optimal** | **14.3** | **grams/day** | **25-34** |  |  |  | **1.3** |
|  | **mean consumption** | **46.9** | **grams/day** | **35-44** |  |  |  | **1.29** |
|  |  |  |  | **45-54** |  |  |  | **1.24** |
|  |  |  |  | **55-64** |  |  |  | **1.19** |
|  |  |  |  | **65-74** |  |  |  | **1.16** |
|  |  |  |  | **≥75** |  |  |  | **1.09** |
| **Processed meats, grams/day** | |  |  |  |  |  |  |  |
|  | **units of RR** | **50** | **grams/day** | **Age** | **RR CHD** | **RR Is Stroke** | **RR He Stroke** | **RR Diabetes** |
|  | **optimal** | **0** | **grams/day** | **25-34** | **1.62** |  |  | **1.86** |
|  | **mean consumption** | **30.7** | **grams/day** | **35-44** | **1.58** |  |  | **1.81** |
|  |  |  |  | **45-54** | **1.47** |  |  | **1.65** |
|  |  |  |  | **55-64** | **1.38** |  |  | **1.52** |
|  |  |  |  | **65-74** | **1.3** |  |  | **1.41** |
|  |  |  |  | **≥75** | **1.16** |  |  | **1.22** |
| **SSBs, 8-oz servings/d** | **BMI (baseline BMI <25)** |  |  |  |  |  |  |  |
|  | **units of RR** | **1** | **8-oz servings/d** | **Age** | **kg/m2** |  |  |  |
|  | **optimal** | **0** | **8-oz servings/d** | **25-34** | **0.1** |  |  |  |
|  | **mean consumption** | **1.0** | **8-oz servings/d** | **35-44** | **0.1** |  |  |  |
|  |  |  |  | **45-54** | **0.1** |  |  |  |
|  |  |  |  | **55-64** | **0.1** |  |  |  |
|  |  |  |  | **65-74** | **0.1** |  |  |  |
|  |  |  |  | **≥75** | **0.1** |  |  |  |
| **SSBs, 8-oz servings/d** | **BMI (baseline BMI >=25)** |  |  |  |  |  |  |  |
|  | **units of RR** | **1** | **8-oz servings/d** | **Age** | **kg/m2** |  |  |  |
|  | **optimal** | **0** | **8-oz servings/d** | **25-34** | **0.23** |  |  |  |
|  | **mean consumption** | **1.0** | **8-oz servings/d** | **35-44** | **0.23** |  |  |  |
|  |  |  |  | **45-54** | **0.23** |  |  |  |
|  |  |  |  | **55-64** | **0.23** |  |  |  |
|  |  |  |  | **65-74** | **0.23** |  |  |  |
|  |  |  |  | **≥75** | **0.23** |  |  |  |
| **SSBs, 8-oz servings/d** | |  |  |  |  |  |  |  |
|  | **units of RR** | **1** | **8-oz servings/d** | **Age** | **RR CHD** | **RR Is Stroke** | **RR He Stroke** | **RR Diabetes (BMI adjusted)** |
|  | **optimal** | **0** | **8-oz servings/d** | **25-34** | **1.33** |  |  | **1.35** |
|  | **mean consumption** | **1.0** | **8-oz servings/d** | **35-44** | **1.31** |  |  | **1.33** |
|  |  |  |  | **45-54** | **1.26** |  |  | **1.27** |
|  |  |  |  | **55-64** | **1.21** |  |  | **1.22** |
|  |  |  |  | **65-74** | **1.17** |  |  | **1.18** |
|  |  |  |  | **≥75** | **1.09** |  |  | **1.1** |
| **SSBs, 8-oz servings/d** | |  |  |  |  |  |  |  |
|  | **units of RR** | **5** | **kg/m2 increase in BMI** | **Age** | **RR CHD** | **RR Stroke** | **RR Diabetes** |  |
|  | **optimal** | **0** | **8-oz servings/d** | **25-34** | **1.45** | **1.24** | **3.55** |  |
|  | **mean consumption** | **1.0** | **8-oz servings/d** | **35-44** | **1.42** | **1.23** | **3.07** |  |
|  |  |  |  | **45-54** | **1.35** | **1.19** | **2.66** |  |
|  |  |  |  | **55-64** | **1.28** | **1.16** | **2.32** |  |
|  |  |  |  | **65-74** | **1.23** | **1.13** | **2.03** |  |
|  |  |  |  | **≥75** | **1.13** | **1.07** | **1.52** |  |
| **PUFA replacing Carbs** | |  |  |  |  |  |  |  |
|  | **units of RR** | **5** | **% energy/day** | **Age** | **RR CHD** | **RR Is Stroke** | **RR He Stroke** | **RR Diabetes** |
|  | **optimal** | **11** | **% energy replacing SFA** | **25-34** | **0.86** |  |  |  |
|  | **mean consumption** | **7.8** | **% energy replacing SFA** | **35-44** | **0.86** |  |  |  |
|  |  |  |  | **45-54** | **0.88** |  |  |  |
|  |  |  |  | **55-64** | **0.9** |  |  |  |
|  |  |  |  | **65-74** | **0.92** |  |  |  |
|  |  |  |  | **≥75** | **0.95** |  |  |  |
| **PUFA replacing SFA** | |  |  |  |  |  |  |  |
|  | **units of RR** | **5** | **% energy/day** | **Age** | **RR CHD** | **RR Is Stroke** | **RR He Stroke** | **RR Diabetes** |
|  | **optimal** | **11** | **% energy replacing SFA** | **25-34** | **0.87** |  |  |  |
|  | **mean consumption** | **7.8** | **% energy replacing SFA** | **35-44** | **0.87** |  |  |  |
|  |  |  |  | **45-54** | **0.89** |  |  |  |
|  |  |  |  | **55-64** | **0.91** |  |  |  |
|  |  |  |  | **65-74** | **0.92** |  |  |  |
|  |  |  |  | **≥75** | **0.96** |  |  |  |
| **Seafood omega3** | |  |  |  |  |  |  |  |
|  | **units of RR** | **100** | **mgrams/day** | **Age** | **RR CHD** | **SBP blacks (ad) (mmHg)** | **SBP HTN (ad) (mmHg)** |  |
|  | **optimal** | **250** | **mgrams/day** | **25-34** | **0.79** | **2.49** | **1.87** |  |
|  | **mean consumption** | **98.1** | **mgrams/day** | **35-44** | **0.8** | **2.49** | **1.87** |  |
|  |  |  |  | **45-54** | **0.82** | **2.49** | **1.87** |  |
|  |  |  |  | **55-64** | **0.85** | **2.49** | **1.87** |  |
|  |  |  |  | **65-74** | **0.87** | **2.49** | **1.87** |  |
|  |  |  |  | **≥75** | **0.93** | **2.49** | **1.87** |  |
| **Sodium, mgrams/day** | |  |  |  |  |  |  |  |
|  | **units of RR** | **2300** | **mgrams/day** | **Age** | **SBP (mmHg)** | **RR Is Stroke** | **RR He Stroke** | **RR Diabetes** |
|  | **optimal** | **2000** |  | **25-34** | **1.64** | **2.3** | **2.25** |  |
|  | **mean consumption** | **3481.5** |  | **35-44** | **2.69** | **2.05** | **2.11** |  |
|  |  |  |  | **45-54** | **3.74** | **1.83** | **1.89** |  |
|  |  |  |  | **55-64** | **4.79** | **1.63** | **1.66** |  |
|  |  |  |  | **65-74** | **5.84** | **1.44** | **1.46** |  |
|  |  |  |  | **≥75** | **5.84** | **1.17** | **1.19** |  |
| **Sodium, mgrams/day** | |  |  |  |  |  |  |  |
|  | **units of RR** | **10** | **mmHg** | **Age** | **RR CHD** |  |  |  |
|  | **optimal** | **2000** |  | **25-34** | **1.81** |  |  |  |
|  | **mean consumption** | **3481.5** |  | **35-44** | **1.68** |  |  |  |
|  |  |  |  | **45-54** | **1.56** |  |  |  |
|  |  |  |  | **55-64** | **1.45** |  |  |  |
|  |  |  |  | **65-74** | **1.33** |  |  |  |
|  |  |  |  | **≥75** | **1.18** |  |  |  |
