# Supplementary material for: Cardiometabolic disease costs associated with suboptimal diet in the United States: A cost analysis based on a microsimulation model
Source: PLoS Med. 2019 Dec 17;16(12):e1002981. doi: 10.1371/journal.pmed.1002981 (PMC6917211; doi:10.1371/journal.pmed.1002981)
Supplement: S3 Text — (DOCX) [file pmed.1002981.s003.docx]

**S3 Text. Detailed information on the ten food/nutrients assessed**

The ten food/nutrients assessed were: (1) fruits: total fruits (grams/day), excluding fruit juices and salted or pickled fruits; (2) vegetables: total vegetables (grams/day), including beans/legumes, and excluding salted, pickled, starchy vegetables (e.g., potatoes, corn) vegetables, or vegetable juices; (3) nuts/seeds: total nuts and seeds (grams/day); (4) whole grains: total whole grain foods and grain‐based products made with 100% whole grains or their flours (grams/day); (5) red meats: total red meats (grams/day), excluding poultry, fish, eggs, and all processed meats; (6) processed meats: total processed meat intake (grams/day); (7) sugar‐sweetened beverages (SSBs): total SSBs (servings [8 fluid ounces]/d) with ≥50 kcal per 8 oz (237 mL) serving, including carbonated soft drinks, fruit drinks, presweetened iced teas, sports drinks, energy drinks, and excluding 100% fruit and vegetable juices, non‐caloric artificially‐sweetened drinks, and alcoholic beverages (servings/d); (8) polyunsaturated fats (PUFA): total PUFA intake (%E/d); saturated fats (SFA): total SFA intake (%E/d); carbohydrates (carbs): total carbohydrate intake (%E/d); (9) seafood omega‐3 fats: total dietary eicosapentaenoic (EPA) + docosahexaenoic (DHA) (mgrams/day) intake, excluding supplements; (10) sodium: total dietary sodium intake (mgrams/day). All dietary factors were adjusted for energy intake (using the residual method (2,000 kcal/d)(1) or, for polyunsaturated fats, as % energy) to reduce measurement error and account for potential differences in body size, lean mass, metabolic efficiency, and physical activity.

1. Willett WC. Correction for the effects of measurement error. Nutritional Epidemiology. 3rd ed. Oxford, England: Oxford University Press; 2013. p. 287-304.
