# Supplementary material for: Cardiometabolic disease costs associated with suboptimal diet in the United States: A cost analysis based on a microsimulation model
Source: PLoS Med. 2019 Dec 17;16(12):e1002981. doi: 10.1371/journal.pmed.1002981 (PMC6917211; doi:10.1371/journal.pmed.1002981)
Supplement: S4 Text — (DOCX) [file pmed.1002981.s004.docx]

**S4 Text. Description of health insurance stratification**

Ten types of insurance categories are reported in the survey data: private, Medicare, Medi-gap, Medicaid, SCHIP (Children’s Health Insurance Program), Military Health Care, State-sponsored health plan, other government program (unspecified), single service plan, or no coverage. Respondents could also report that they did not know what insurance coverage they had, and those were categorized as other coverage. We consolidated the insurance responses into 6 categories: (1) private (private; single service plan; private plus other government; other coverage); (2) Medicare (Medicare; Medi-Gap; Medicare plus other government; Medicare plus private); (3) Medicaid; (4) Dual eligible (Medicare plus Medicaid); (5) other government (other government; state-sponsored; military); and (6) no coverage. Due the limited number of individuals in the survey from SCHIP category, those were excluded from the analyses. The insurance responses were merged into these categories according to the overall characteristic of the insurance type and the mean income levels of individuals from each insurance group. Modeled population description and dietary intake by health insurance are provided in S3 Table and S4 Table.
