# Supplementary material for: Cardiometabolic disease costs associated with suboptimal diet in the United States: A cost analysis based on a microsimulation model
Source: PLoS Med. 2019 Dec 17;16(12):e1002981. doi: 10.1371/journal.pmed.1002981 (PMC6917211; doi:10.1371/journal.pmed.1002981)
Supplement: S5 Text — (DOCX) [file pmed.1002981.s005.docx]

**S5 Text. Attributing Proportion of Healthcare Costs by Consumer Category and Ultimate Cost-Bearer**

In this study, we recognize that consumers, government, and the private sector are the ultimate cost-bearers of healthcare. For example, even when the proximate source of payment for a medical bill is an insurance company, the ultimate cost-bearers are the consumer, private sector, or government program that paid the insurance premiums. Therefore, a dietary intervention that result in healthcare cost savings ultimately benefit the consumers (through reduced out-of-pocket costs and health insurance premiums), the government (through lower public medical insurance program costs), and to other private sector entities (mostly to employers through reductions in employers’ portion of health insurance premiums, but also to charitable entities, and hospitals that provide medical services, etc).

In order to attribute total healthcare cost savings to their ultimate cost-bearers, we estimated the proportion of healthcare costs contributed by household, government and the private sector, using data from the Centers for Medicare and Medicaid Services (CMS) and the Medical Expenditure Survey (MEPS).

The proportions of healthcare costs contributed by their ultimate cost-bearers, stratified by consumer category (e.g. insurance status), are shown in S5**Table**. Below are the four steps we used to estimate these proportions:

1. Locate data from MEPS that showed the proportions of medical expenses paid by various sources of payments, stratified by insurance status.(1) Source of payments referred to out-of-pocket payments, private insurance payments, Medicare payments, Medicaid payments, and other payments.
2. Because data from MEPS were only suitable for 4 of our consumer categories (private, Medicare, dual-eligible, and no coverage), we searched for additional literature to estimate the proportion of medical expenses paid by various sources of payments for Medicaid category and the other government insurance category. Details below.
3. We determined the ultimate cost-bearers for each source of payment based on CMS data.(2, 3)
4. For out-of-pocket payments, households are the sole cost-bearer.
5. For Medicaid payments, government is the sole cost-bearer.
6. Households, government, and businesses are all cost bearers of private insurance, in proportions that were estimated in step #1 above.
7. Traditionally in CMS literature, households, government, and business are all cost-bearers of Medicare, as household and businesses contribute to Medicare through payroll taxes. However, in our calculation, we attributed expenses funded by Medicare payroll taxes as government expenses, as we believe healthcare cost saving from a dietary intervention will not likely result in a reduction in Medicare payroll taxes. Therefore, in our calculation, government is deemed the sole cost-bearer of Medicare payments. Essentially, we treat Medicare as a government medical program, more similar to Medicaid than to a private insurance program.
8. Other insurance policies are paid for by government (e.g. VA insurance) and businesses (e.g. workers’ compensation).
9. Because private insurance and other insurances are paid by multiple cost-bearers, we estimated the percentages of household, government and business contribution to private insurance and other insurances using CMS data.(2, 3) S6 **Table** shows 2014 total U.S. personal health expenditure by sources of payments and ultimate cost-bearers. Based on S6 **Table**, we estimated that the percentages of household, government and business contribution to private insurance are 31.96%, 19.9%, and 48.14%, respectively. For a mix of other insurance policies, government contributes 36.14%, and businesses contribute 63.87% (not shown).

**Cost-Bearer Estimation for Medicaid Category**

Due to lack of data from MEPS, we estimated the proportions of Medicaid expenses for Medicaid participants using other literature. CMS's 2015 Actuarial Report estimated that spending per Medicaid enrollee in 2014 was $7,315.(4) Out-of-Pocket expenses for Medicaid participants were estimated to be roughly $300 using a Center for Medicare Advocacy report(5) and a 2014 New York Times article(6) citing Kaiser Family Foundation data. Using these sources, we assumed that Medicaid households would be responsible for roughly 5% of their medical expenses through out-of-pocket payments, and the government would the bear the cost of the rest (S5 Table).

**Cost-Bearer Estimation for Other Government Insurance Category**

Due to difference in MEPS insurance classification and our own classification using NHANES, we could not directly use MEPS for the proportions of medical expenses paid by various sources of payments for those consumers that we classified in the “other government” category. Therefore, we assumed that the proportion of expenses would be similar to consumers who are primarily on private insurance. However, we attributed to government the portion of private insurance payments paid by employers. In short, as with consumers in the Private Insurance category, consumers in the Other Government category contribute 38.2% of their medical expenses **(S5 Table)**. However, government pays a higher proportion of the medical expenses for those in the Other Government category, compared to those on the Private Insurance category **(S5 Table)**.

**Additional Assumptions**

1. CMS distinguishes between federal government and state, local government as different cost-bearers; in our analysis, we summed them into one cost-bearer which we referred to as “government.”
2. CMS distinguishes between businesses and third-party payers (e.g. health-related philanthropic support, nonoperating revenue, investment income, and privately-funded structures and equipment); we summed them into one group that we referred to as “other private sector” in the manuscript.
3. Data from MEPS are the proportions of medical expenses paid by various sources of payments were derived from for expenses for all health services. We assumed that the proportions would be similar for expenses specific to cardiovascular events.

For attributing the percentages of household, government and business contribution to private insurance and other insurances, we used CMS’ data on US Personal Healthcare Expenditure (PHE). An alternative that we could have used is to the US National Health Expenditures (NHE).(7) PHE include spending for hospital care, physicians and clinics' services, dentists' services, drugs, nursing home care, and other residential, home and personal care. NHE measure the total costs of health care in the United States; therefore it includes PHE, as well as government administration cost, net cost of private health insurance, public health activities, and investment.(7) We chose PHE, as healthcare cost savings as a result of a dietary intervention would largely be reflected in PHE.

1. Medical Expenditure Panel Survey. Table 1: Total Health Services-Median and Mean Expenses per Person With Expense and Distribution of Expenses by Source of Payment: United States, 2014. 2014; Available from: https://meps.ahrq.gov/mepsweb/data_stats/tables_compendia_hh_interactive.jsp?_SERVICE=MEPSSocket0&_PROGRAM=MEPSPGM.TC.SAS&File=HCFY2014&Table=HCFY2014_PLEXP_%40&VAR1=AGE&VAR2=SEX&VAR3=RACETH5C&VAR4=INSURCOV&VAR5=POVCAT14&VAR6=REGION&VAR7=HEALTH&VARO1=4+17+44+64&VARO2=1&VARO3=1&VARO4=1&VARO5=1&VARO6=1&VARO7=1&_Debug=.

2. Centers for Disease Control and Prevention. Table 95 (page 1 of 3). Personal health care expenditures, by source of funds and type of expenditure: United States, selected years 1960–2014.

3. Centers for Disease Control and Prevention. Table 100 (page 1 of 2). National health expenditures and percent distribution, by sponsor: United States, selected years 1987–2014.

4. Centers for medicaid and Medicare Services. 2015 Actuarial report on the financial outlook for Medicaid. 2015.

5. Center for Medicare Advocacy. Medicare Cost-Sharing for Dual Eligibles: Who Pays What for Whom? 2005.

6. Carroll AE. Medicaid Gives the Poor a Reason to Say No Thanks. The New York Times. 2014.

7. Centers for medicaid and Medicare Services. National Health Expenditure Accounts: Methodology Paper, 2014 Definitions, Sources, and Methods. 2014.
