# Supplementary material for: Cardiometabolic disease costs associated with suboptimal diet in the United States: A cost analysis based on a microsimulation model
Source: PLoS Med. 2019 Dec 17;16(12):e1002981. doi: 10.1371/journal.pmed.1002981 (PMC6917211; doi:10.1371/journal.pmed.1002981)
Supplement: S6 Text — (DOCX) [file pmed.1002981.s006.docx]

**S6 Text. Modeled costs estimates**

For diabetes costs estimation, at every timestep in the model, individuals without diabetes have a chance to become diabetic. The probability of developing diabetes is based on age and time incidence trends from NHANES. This baseline probability can be reduced if an individual is on a diabetes-specific intervention. A corresponding annual chronic diabetes cost ($5,505) is added to all diabetic individuals while alive(1, 2).

The model used published relative risk values by age showing the effect of the 10 foods/nutrients consumption on coronary heart disease (CHD), stroke, and diabetes(3). For each food/nutrient unit change in consumption specific relative risks were applied on their effects over the health outcome, modified by age. For example, if a 50-year old individual increases consumption of fruits in 100g the associated risk reduction in CHD will be 7%. The only exception is sodium, in which BP reductions per change in sodium consumption came from the same source(3) but the effect BP has on CHD and stroke came from reductions in relative risks from the US based Framingham risk equation that were already built in the model(4). Further details about the relative risk estimates are provided in S7 Table. Model outputs included predicted estimates of CVD events, CVD mortality rates and event-associated health care costs. Five years estimates were generated (S 8-11 Tables) and annual count of CVD events (S12 and S13 Tables) and costs were calculated based on the five years results. The option to estimate the one year cost based on the five years run of the model was made under the assumption that the chronic costs would be better captured in a longer model run, when compared to a one year run.

1. Economic costs of diabetes in the U.S. in 2012. Diabetes Care. 2013;36(4):1033-46.

2. Zhuo X, Zhang P, Hoerger TJ. Lifetime Direct Medical Costs of Treating Type 2 Diabetes and Diabetic Complications. American Journal of Preventive Medicine. 2013;45(3):253-61.

3. Micha R, Peñalvo JL, Cudhea F, Imamura F, Rehm CD, Mozaffarian D. Association Between Dietary Factors and Mortality From Heart Disease, Stroke, and Type 2 Diabetes in the United States. Jama. 2017;317(9):912-24.

4. Pandya A, Sy S, Cho S, Alam S, Weinstein MC, Gaziano TA. Validation of a Cardiovascular Disease Policy Micro-Simulation Model using Both Survival and Receiver Operating Characteristic Curves. Medical decision making : an international journal of the Society for Medical Decision Making. 2017:272989x17706081.
