# Supplementary material for: Cardiometabolic disease costs associated with suboptimal diet in the United States: A cost analysis based on a microsimulation model
Source: PLoS Med. 2019 Dec 17;16(12):e1002981. doi: 10.1371/journal.pmed.1002981 (PMC6917211; doi:10.1371/journal.pmed.1002981)
Supplement: S1 Fig — CVD PREDICT, Cardiovascular Disease Policy Model for Risk, Events, Detection, Interventions, Costs, and Trends. (DOCX) [file pmed.1002981.s007.docx]

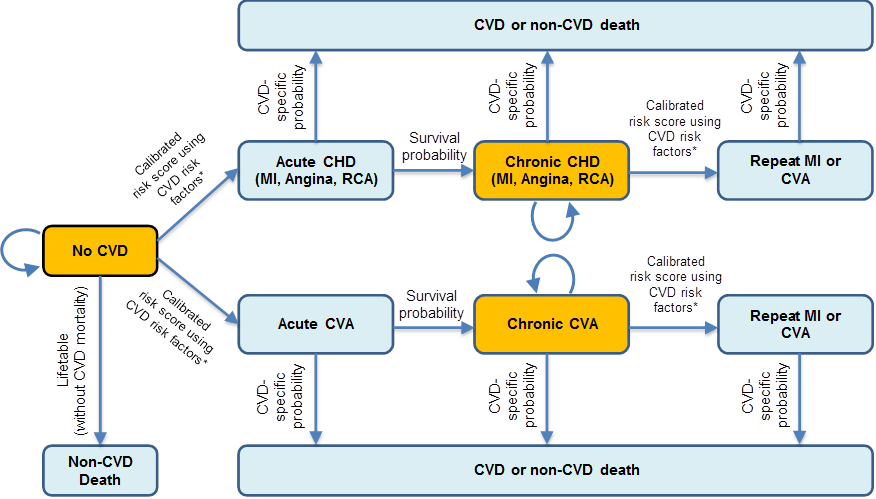


S1 Fig. The CVD-PREDICT microsimulation model.

CVD – cardiovascular disease, CVA – cerebrovascular accident, MI – myocardial infarction, RCA – resuscitated cardiac arrest

Transitions were based on a calibrated risk score including age, sex, systolic blood pressure, total cholesterol, HDL cholesterol, current smoking, and diabetes status. Baseline risk factors were derived from NHANES 2009-12, with further annual changes in all risk factors incorporating both age and secular trends.
