# Supplementary material for: Cardiometabolic disease costs associated with suboptimal diet in the United States: A cost analysis based on a microsimulation model
Source: PLoS Med. 2019 Dec 17;16(12):e1002981. doi: 10.1371/journal.pmed.1002981 (PMC6917211; doi:10.1371/journal.pmed.1002981)
Supplement: S2 Fig — (DOCX) [file pmed.1002981.s008.docx]

**CMD Costs**

**Associated with**

**Suboptimal dietary**

**Habits**

**US$ 50,443 M**

Acute US$ 42,567 M

Chronic US$ 7,206 M

Drug-related US$ 670 M


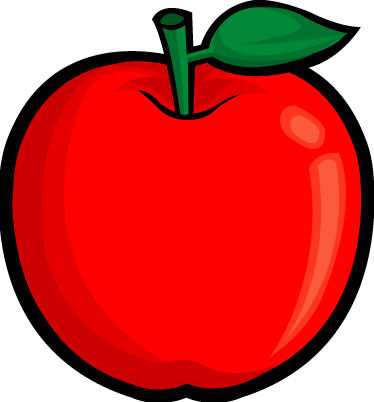

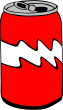

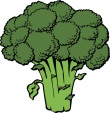

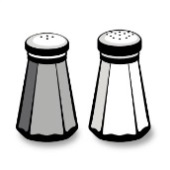

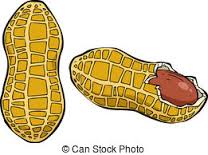

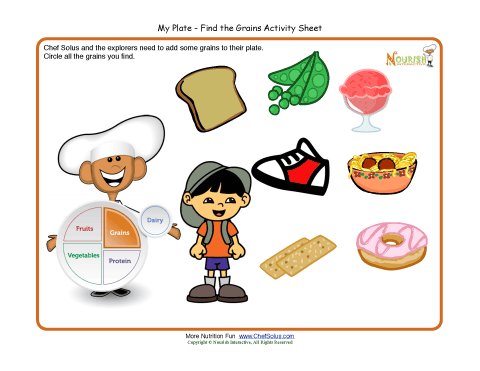

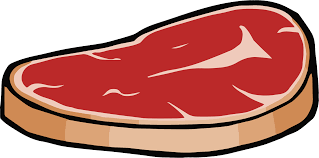

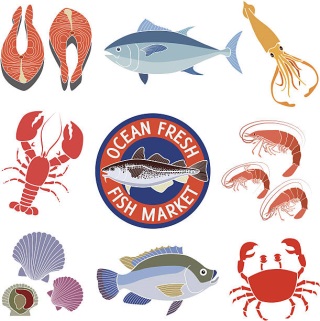

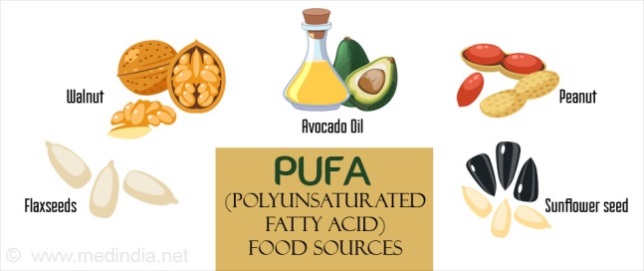

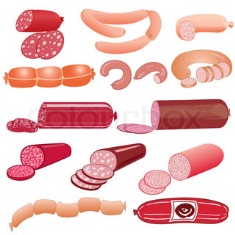


CHD

Stroke

300 g/day

CHD

Stroke

400 g/day

CHD

Diabetes

20 g/day

CHD

Diabetes

Stroke

125 g/day

Diabetes

14.3 g/day

CHD

Diabetes

0 g/day

CHD

Diabetes

Stroke

0 oz/day

CHD

11% Energy/day

CHD

250 mg/day

Systolic BP

2000 mg/day

US$ 12,736 M

US$ 3,854 M

US$ 9,552 M

US$ 10,055 M

US$ 13,574 M

US$ 7,541 M

US$ 503 M

US$ 10,223 M

US$ 9,720 M

US$ 3,352 M

**S2 Fig. Pathways for cardiometabolic disease prevention associated with optimal dietary habits** **and annual Cardiometabolic Costs Among the US Adult (Aged ≥35 years) Population Associated with Suboptimal Dietary Habits.**

Values given in 2018 US millions of dollars. Total cost does not reflect sum of individual components based on the assumption that the benefits of the 10 food groups are not independent.

Clockwise from top: Fruits, Vegetables, Nuts/Seeds, Whole grains, Red meat, Sugar-sweetened beverages, Processed meat, Polyunsaturated fatty acids, seafood omega-3, Sodium.

Color of arrow indicates direction of benefit (green: increase consumption for more benefit; red: decrease consumption for more benefit).

CHD – coronary heart disease; BP – blood pressure
