# Supplementary material for: Cardiometabolic disease costs associated with suboptimal diet in the United States: A cost analysis based on a microsimulation model
Source: PLoS Med. 2019 Dec 17;16(12):e1002981. doi: 10.1371/journal.pmed.1002981 (PMC6917211; doi:10.1371/journal.pmed.1002981)
Supplement: S3 Fig — (DOCX) [file pmed.1002981.s009.docx]

| **S3 Fig. Annual Ultimate Cost-Bearer of Cardiometabolic Costs Among US Adults Aged ≥35 years**  **Associated with Suboptimal Dietary Habits, by Health Insurance Type.**  Values given in US dollars.  Private includes: private; single service plan; private plus other government; other coverage.  Medicare includes: Medicare; Medi-Gap; Medicare plus other government; Medicare plus private.  Medicaid includes only Medicaid.  Dual eligible includes: Medicare plus Medicaid.  Other government includes: other government; state-sponsored; military.  Household includes out of pocket expenses and/or premiums which includes household contribution to employer-sponsored insurance  premiums, direct purchase of insurance, and medical portion of property and casualty insurance. |
| --- |
| Third party includes employers (e.g. employer sponsored health insurance, worksite health, workers' compensation),  hospitals, nonprofits, health-related philanthropic support. |
