# Supplementary material for: Cardiometabolic disease costs associated with suboptimal diet in the United States: A cost analysis based on a microsimulation model
Source: PLoS Med. 2019 Dec 17;16(12):e1002981. doi: 10.1371/journal.pmed.1002981 (PMC6917211; doi:10.1371/journal.pmed.1002981)
Supplement: S1 Table — CVD PREDICT, Cardiovascular Disease Policy Model for Risk, Events, Detection, Interventions, Costs, and Trends. (DOCX) [file pmed.1002981.s010.docx]

**S1 Table. Costs used in the CVD PREDICT model Parameters**

| Parameter | **Base-Case Value** | **Base-Case Source** |
| --- | --- | --- |
| ***Costs for Chronic Disease States*** |  |  |
| Disease Free | $0 | Assumption: None |
| Chronic CHD^a^ | $3,201 | Lee 2010 (1) |
| Chronic Stroke | $2,115 | Pignone 2006 (2) |
| ***Costs for Acute Disease States*** | | |
| Acute Cardiac Arrest | $19,271 | O'Sullivan 2011 (3) |
| Acute MI^b^ | $56,358 | O'Sullivan 2011 (3) |
| Acute Angina | $29,139 | O'Sullivan 2011 (3) |
| Acute Stroke | $19,128 | O'Sullivan 2011 (3) |
| ***Costs for Procedures and Repeat Events*** | | |
| Repeat MI^b^ | $56,358 | O'Sullivan 2011 (3) |
| Repeat Stroke | $19,128 | O'Sullivan 2011 (3) |
| CABG^c^ | $36,872 | O'Sullivan 2011 (3) |
| PTCA^d^ | $34,742 | O'Sullivan 2011 (3) |
| ***Screening Costs*** | | |
| Non-lab test (GP^e^ visit in Stage 1) | $75 | Pletcher 2009 (4) |
| Cholesterol (lab) test | $35 | Pletcher 2009 (4) |
| # extra GP^e^ visits during Stage 2 | 1 | Assumption |
| # lab tests/year after treatment | 1 | Lazar 2011(5), Expert Opinion |
| # GP^e^ visits/year after treatment | 1 | Lazar 2011(5), Expert Opinion |
| ***Statin Drug and Adverse Event Costs*** | | |
| Statin | $267 | Redbook 2009 (6) |
| Anti-hypertensive | $206 | Nuckols 2011 (7) |
| Aspirin | $7.6 | Pignone 2006 (2) |
| ACE Inhibitor | $52.12 | Shah 2011(8), Redbook 2009 (6) |
| Beta Blocker | $52.12 | Shah 2011(8), Redbook 2009 (6) |
| Mild adverse event | $178.53 | Lee 2010 (1) |
| Major adverse event | $7,033 | Lee 2010 (1) |

^a^Coronary heart disease; ^b^Myocardial infarction; ^c^Coronary artery bypass graft; ^d^Percutaneous transluminal coronary angioplasty; ^e^General practitioner.

1. Lee KK, Cipriano LE, Owens DK, Go AS, Hlatky MA. Cost-effectiveness of using high-sensitivity C-reactive protein to identify intermediate- and low-cardiovascular-risk individuals for statin therapy. Circulation. 2010;122(15):1478-87. Epub 2010/09/30.

2. Pignone M, Earnshaw S, Tice JA, Pletcher MJ. Aspirin, statins, or both drugs for the primary prevention of coronary heart disease events in men: a cost-utility analysis. Ann Intern Med. 2006;144(5):326-36. Epub 2006/03/08.

3. O'Sullivan AK, Rubin J, Nyambose J, Kuznik A, Cohen DJ, Thompson D. Cost estimation of cardiovascular disease events in the US. Pharmacoeconomics. 2011;29(8):693-704. Epub 2011/05/19.

4. Pletcher MJ, Lazar L, Bibbins-Domingo K, Moran A, Rodondi N, Coxson P, et al. Comparing impact and cost-effectiveness of primary prevention strategies for lipid-lowering. Ann Intern Med. 2009;150(4):243-54. Epub 2009/02/18.

5. Lazar LD, Pletcher MJ, Coxson PG, Bibbins-Domingo K, Goldman L. Cost-effectiveness of statin therapy for primary prevention in a low-cost statin era. Circulation. 2011;124(2):146-53. Epub 2011/06/29.

6. Thomson Healthcare. Red book Montvale, NJ: Thomson PDR; 2014.

7. Nuckols TK, Aledort JE, Adams J, Lai J, Go M-H, Keesey J, et al. Cost Implications of Improving Blood Pressure Management among U.S. Adults. Health Services Research. 2011;46(4):1124-57.

8. Shah ND, Mason J, Kurt M, Denton BT, Schaefer AJ, Montori VM, et al. Comparative Effectiveness of Guidelines for the Management of Hyperlipidemia and Hypertension for Type 2 Diabetes Patients. PLoS ONE. 2011;6(1):e16170.
