# Supplementary material for: Cardiometabolic disease costs associated with suboptimal diet in the United States: A cost analysis based on a microsimulation model
Source: PLoS Med. 2019 Dec 17;16(12):e1002981. doi: 10.1371/journal.pmed.1002981 (PMC6917211; doi:10.1371/journal.pmed.1002981)
Supplement: S2 Table — (DOCX) [file pmed.1002981.s011.docx]

**S2 Table. Model Population description for Adults aged 35 years**

| **Variables** | **Mean or proportion** | **Standard Deviation** |  |
| --- | --- | --- | --- |
| **Age** (years) | 37.2 | 1.4 |  |
| **Female** (%) | 52.0 |  |  |
| **White** (%) | 60.8 |  |  |
| **African Americans** (%) | 10.6 |  |  |
| **Hispanic** (%) | 19.2 |  |  |
| **<High school** (%) | 17.6 |  |  |
| **High school** (%) | 43.6 |  |  |
| **College** (%) | 38.8 |  |  |
| **Body mass index** (kg/m2) | 28.9 | 6.7 |  |
| **Systolic blood pressure** (mmHg) | 115.6 | 13.8 |  |
| **Diastolic blood pressure** (mmHg) | 72.2 | 11.3 |  |
| **Total Cholesterol** (mgrams/L) | 196.9 | 41.7 |  |
| **HDL-cholesterol** (mgrams/L) | 51.6 | 15.0 |  |
| **LDL-cholesterol** (mgrams/L) | 116.8 | 35.7 |  |
| **Triglycerides** (mgrams/L) | 120.3 | 96.7 |  |
| **History of Diabetes** (%) | 2.8 |  |  |
| **Current Smoker** (%) | 19.3 |  |  |
| **Current Hypertension Treatment** (%) | 12.1 |  |  |
| **Angina** (%) | 0.2 |  |  |
| **Myocardial Infarction** (%) | 0.4 |  |  |
| **Stroke** (%) | 0.3 |  |  |
|  | **Mean or proportion** | **Standard Deviation** | **Optimal^a^ (%)** |
| **Fruits Excluding Fruit Juices,** grams/day | 97.5 | 133.6 | 6.1 |
| **Vegetables Including Legumes,** grams/day | 188.4 | 142.5 | 8.4 |
| **Nuts/Seeds,** grams/day | 10.0 | 20.0 | 16.3 |
| **Whole Grains,** grams/day | 20.6 | 27.2 | 0.8 |
| **Red Meats, Unprocessed,** grams/day | 49.2 | 53.1 | 33.4 |
| **Processed Meats,** grams/day | 31.6 | 40.1 | 33.9 |
| **Sugar sweetened-beverages,** 8-oz servings/day | 1.5 | 1.9 | 36.4 |
| **PUFAs^b^,** % energy replacing carbohydrates or saturated fats | 7.8 | 2.6 | 10.1 |
| **Seafood Omega-3 Fats,** mgrams/day | 98.4 | 215.5 | 7.7 |
| **Sodium,** mgrams/day | 3423.7 | 873.0 | 2.8 |

^a^Percentage of individuals with optimal or better than optimal consumption of the dietary item (Fruits excluding fruit juices: 300grams/day; Vegetables including legumes: 400grams/day; Nuts/seeds: 20.2 grams/day (5 1-oz servings/wk); Whole grains: 125grams/day (2.5 50-g servings/d); Red meats, unprocessed: 14.3 grams/day (1 100-g serving/wk); Processed meats: No intake; SSBs: No intake; PUFAs: 11% energy replacing carbohydrates or saturated fats; Seafood omega-3 fats: 250mgrams/day; Sodium: 2000mgrams/day).

^b^PUFAs – polyunsaturated fatty acids
