# Supplementary material for: Cardiometabolic disease costs associated with suboptimal diet in the United States: A cost analysis based on a microsimulation model
Source: PLoS Med. 2019 Dec 17;16(12):e1002981. doi: 10.1371/journal.pmed.1002981 (PMC6917211; doi:10.1371/journal.pmed.1002981)
Supplement: S3 Table — (DOCX) [file pmed.1002981.s012.docx]

| **S3 Table. Modeled population description by health insurance^a^** | | | | | | | | | | | | | |
| --- | --- | --- | --- | --- | --- | --- | --- | --- | --- | --- | --- | --- | --- |
|  | Private | | | Medicare | | Medicaid | | Dual Eligible | | Other Government | | No Coverage | |
| **N** | 93,344,765 |  | | 32,679,047 |  | 5,530,300 |  | 2,178,603 |  | 8,881,997 |  | 24,970,144 |  |
| **(%)** | 55.7 |  | | 19.5 |  | 3.3 |  | 1.3 |  | 5.3 |  | 14.9 |  |
|  | Mean or proportion | Standard Deviation | Mean or proportion | | Standard Deviation | Mean or proportion | Standard Deviation | Mean or proportion | Standard Deviation | Mean or proportion | Standard Deviation | Mean or proportion | Standard Deviation |
| **Age** (years) | 51.2 | 10.0 | 71.5 | | 7.9 | 49.1 | 10.5 | 62.7 | 11.7 | 52.3 | 10.5 | 48.6 | 9.0 |
| **Female** (%) | 51.9 |  | 55.1 | |  | 65.9 |  | 66.0 |  | 54.6 |  | 47.9 |  |
| **White** (%) | 78.6 |  | 81.8 | |  | 34.2 |  | 51.1 |  | 64.4 |  | 49.0 |  |
| **African Americans** (%) | 8.0 |  | 8.4 | |  | 29.7 |  | 22.0 |  | 13.0 |  | 13.9 |  |
| **Hispanic** (%) | 7.5 |  | 6.1 | |  | 27.6 |  | 18.3 |  | 15.3 |  | 29.5 |  |
| **<High school** (%) | 9.0 |  | 23.6 | |  | 39.3 |  | 43.5 |  | 18.6 |  | 33.9 |  |
| **High school** (%) | 48.9 |  | 55.8 | |  | 52.0 |  | 54.3 |  | 47.4 |  | 54.2 |  |
| **College** (%) | 42.1 |  | 20.6 | |  | 8.7 |  | 2.2 |  | 34.0 |  | 11.9 |  |
| **Body mass index** (kg/m2) | 29.0 | 6.4 | 28.9 | | 6.5 | 31.6 | 7.9 | 31.9 | 7.3 | 29.7 | 7.0 | 29.4 | 6.6 |
| **Systolic blood pressure** (mmHg) | 120.9 | 15.1 | 131.5 | | 19.7 | 123.6 | 18.6 | 130.9 | 20.2 | 122.2 | 17.5 | 124.5 | 19.1 |
| **Diastolic blood pressure** (mmHg) | 73.1 | 10.5 | 67.6 | | 12.1 | 72.1 | 13.0 | 72.7 | 14.7 | 72.8 | 12.7 | 74.2 | 12.2 |
| **Total Cholesterol** (mgrams/L) | 204.9 | 41.6 | 192.7 | | 42.0 | 200.5 | 42.6 | 190.0 | 39.3 | 196.9 | 40.0 | 208.1 | 45.3 |
| **HDL-cholesterol** (mgrams/L) | 54.9 | 17.4 | 55.0 | | 16.4 | 51.6 | 16.0 | 53.2 | 16.7 | 54.2 | 16.3 | 50.4 | 14.6 |
| **LDL-cholesterol** (mgrams/L) | 119.6 | 35.0 | 110.4 | | 35.0 | 122.2 | 40.1 | 103.0 | 39.4 | 110.5 | 32.8 | 130.1 | 40.9 |
| **Triglycerides** (mgrams/L) | 130.3 | 119.7 | 133.2 | | 76.9 | 146.5 | 119.1 | 132.9 | 64.1 | 140.0 | 128.0 | 147.7 | 89.9 |
| **History of Diabetes** (%) | 7.9 |  | 20.1 | |  | 18.6 |  | 32.6 |  | 12.5 |  | 9.2 |  |
| **Current smoker** (%) | 13.3 |  | 10.8 | |  | 38.9 |  | 20.2 |  | 20.8 |  | 29.8 |  |
| **Current hypertension treatment** (%) | 27.9 |  | 58.7 | |  | 46.4 |  | 65.4 |  | 43.5 |  | 23.1 |  |
| **Angina** (%) | 1.2 |  | 6.2 | |  | 2.0 |  | 10.7 |  | 2.5 |  | 2.6 |  |
| **Myocardial infarction** (%) | 1.9 |  | 10.9 | |  | 5.6 |  | 9.9 |  | 4.6 |  | 3.5 |  |
| **Stroke** (%) | 1.4 |  | 8.8 | |  | 5.3 |  | 15.1 |  | 4.5 |  | 1.4 |  |

^a^Health insurance - Private includes: private, single service plan, private plus other government, other coverage; Medicare includes: Medicare, Medi-Gap, Medicare plus other government, Medicare plus private; Medicaid includes only Medicaid; Dual eligible includes: Medicare plus Medicaid; and Other government includes: other government; state-sponsored; military.
