# Supplementary material for: Cardiometabolic disease costs associated with suboptimal diet in the United States: A cost analysis based on a microsimulation model
Source: PLoS Med. 2019 Dec 17;16(12):e1002981. doi: 10.1371/journal.pmed.1002981 (PMC6917211; doi:10.1371/journal.pmed.1002981)
Supplement: S4 Table — (DOCX) [file pmed.1002981.s013.docx]

| **S4 Table.** **Modeled population dietary intake by health insurance^a^** | | | | | | | | | | | | | | | | | | | | | | | | |
| --- | --- | --- | --- | --- | --- | --- | --- | --- | --- | --- | --- | --- | --- | --- | --- | --- | --- | --- | --- | --- | --- | --- | --- | --- |
|  |  | Private | | | | Medicare | | | | Medicaid | | | Dual Eligible | | | | Other Government | | | | No Coverage | | | |
|  |  |  |  | | Optimal^b^ |  |  | | Optimal^b^ |  |  | Optimal^b^ |  |  | | Optimal^b^ |  |  | | Optimal^b^ |  |  | | Optimal^b^ |
|  |  | Mean | | Standard Deviation | % | Mean | | Standard Deviation | % | Mean | Standard Deviation | % | Mean | | Standard Deviation | % | Mean | | Standard Deviation | % | Mean | | Standard Deviation | % |
| **Fruits Excluding Fruit Juices,** grams/day |  | 120.8 | | 150.1 | 9.4 | 137.4 | | 134.8 | 11.1 | 91.4 | 119.5 | 5.6 | 120.0 | | 138.5 | 10.2 | 115.3 | | 144.9 | 6.2 | 106.2 | | 144.6 | 7.8 |
| **Vegetables Including Legumes,** grams/day |  | 199.5 | | 166.1 | 9.2 | 176.6 | | 132.4 | 5.1 | 156.1 | 127.4 | 6.2 | 155.3 | | 120.2 | 3.9 | 187.4 | | 155.8 | 11.5 | 171.6 | | 131.0 | 5.9 |
| **Nuts/Seeds,** grams/day |  | 14.6 | | 30.5 | 23.0 | 12.8 | | 32.4 | 19.5 | 7.9 | 32.0 | 9.0 | 6.4 | | 17.9 | 10.9 | 10.8 | | 19.6 | 19.9 | 6.6 | | 18.2 | 9.8 |
| **Whole Grains,** grams/day |  | 22.7 | | 26.5 | 0.8 | 25.9 | | 27.2 | 0.7 | 15.8 | 23.3 | 0.8 | 16.4 | | 21.5 | 0.6 | 22.2 | | 25.5 | 0.0 | 15.4 | | 22.4 | 0.3 |
| **Red Meats, Unprocessed,** grams/day |  | 45.7 | | 48.8 | 35.9 | 45.1 | | 51.9 | 37.8 | 46.4 | 52.0 | 34.1 | 43.1 | | 48.7 | 41.2 | 49.2 | | 54.7 | 36.7 | 53.4 | | 55.4 | 34.1 |
| **Processed Meats,** grams/day |  | 30.9 | | 38.3 | 31.1 | 30.5 | | 36.4 | 31.8 | 29.5 | 40.7 | 37.0 | 24.7 | | 33.7 | 31.6 | 30.7 | | 38.0 | 35.5 | 31.2 | | 41.0 | 31.8 |
| **Sugar sweetened-beverages,** 8-oz servings/day |  | 0.9 | | 1.5 | 50.4 | 0.7 | | 1.3 | 56.7 | 1.5 | 2.2 | 41.3 | 1.4 | | 1.7 | 42.1 | 1.1 | | 1.8 | 46.3 | 1.5 | | 1.6 | 30.8 |
| **PUFAs^c^,** % energy replacing carbohydrates or saturated fats |  | 8.0 | | 2.7 | 12.3 | 8.0 | | 2.7 | 12.1 | 7.2 | 2.7 | 6.6 | 7.1 | | 2.8 | 13.9 | 7.4 | | 2.8 | 12.2 | 7.2 | | 2.4 | 7.7 |
| **Seafood Omega-3 Fats,** mgrams/day |  | 96.0 | | 173.9 | 8.9 | 99.7 | | 190.6 | 9.5 | 125.9 | 248.1 | 13.8 | 110.5 | | 203.9 | 11.4 | 101.1 | | 201.3 | 8.4 | 95.5 | | 210.2 | 8.7 |
| **Sodium,** mgrams/day |  | 3462.7 | | 878.5 | 1.9 | 3553.7 | | 899.9 | 1.7 | 3527.4 | 1023.3 | 4.5 | 3516.2 | | 906.7 | 1.3 | 3467.7 | | 1002.2 | 6.8 | 3449.1 | | 1282.1 | 3.1 |

^a^Health insurance - Private includes: private, single service plan, private plus other government, other coverage; Medicare includes: Medicare, Medi-Gap, Medicare plus other government, Medicare plus private; Medicaid includes only Medicaid; Dual eligible includes: Medicare plus Medicaid; and Other government includes: other government; state-sponsored; military.

^b^Percentage of individuals with optimal or better than optimal consumption of the dietary item (Fruits excluding fruit juices: 300grams/day; Vegetables including legumes: 400grams/day; Nuts/seeds: 20.2 grams/day (5 1-oz servings/wk); Whole grains: 125grams/day (2.5 50-g servings/d); Red meats, unprocessed: 14.3 grams/day (1 100-g serving/wk); Processed meats: No intake; SSBs: No intake; PUFAs: 11% energy replacing carbohydrates or saturated fats; Seafood omega-3 fats: 250mgrams/day; Sodium: 2000mgrams/day).

^c^PUFAs – polyunsaturated fatty acids
