# Supplementary material for: Cardiometabolic disease costs associated with suboptimal diet in the United States: A cost analysis based on a microsimulation model
Source: PLoS Med. 2019 Dec 17;16(12):e1002981. doi: 10.1371/journal.pmed.1002981 (PMC6917211; doi:10.1371/journal.pmed.1002981)
Supplement: S5 Table — (DOCX) [file pmed.1002981.s014.docx]

| **S5 Table. Distribution of Healthcare Cost by Consumer Category and Ultimate Cost-Bearer^a^** | | | | | | |
| --- | --- | --- | --- | --- | --- | --- |
|  | 6 Consumer Categories | | | | | |
| **Ultimate Cost-Bearers** | **Private** | **Medicare** | **Medicaid^b^** | **Dual Eligible** | **Other Gov.** | **No Coverage** |
| **Household** | 38.20% | 18.02% | 5.00% | 2.70% | 38.20% | 24.20% |
| private insurance^c^ | 23.39% | 4.91% | 0.00% | 0.00% | 23.39% | 0.00% |
| out of pocket | 14.81% | 13.11% | 5.00% | 2.70% | 14.81% | 24.20% |
| **Other Private Sector**^d^ | 38.04% | 11.73% | 0.00% | 4.28% | 2.81% | 48.41% |
| **Government** | 23.76% | 70.25% | 95.00% | 93.02% | 58.99% | 27.39% |
| Total | 100.0% | 100.0% | 100.0% | 100.0% | 100.0% | 100.0% |
| ^a^ Percentages were calculated using data from CMS (eTable 5) and data from the Medical Expenditure Panel Survey. | | | | | | |
| ^b^ Due to lack of data from MEPS, we estimated the distribution of expenses for Medicaid using literature. CMS's 2015 Actuarial Report estimated that spending per Medicaid enrollee cost in 2014 is $7,315. Out-of-Pocket expenses is estimated to be roughly $300 using a 2005 Center on Budget and Policy Priorities report and a 2014 NYTimes article citing Kaiser Family Foundation data. See below for links to sources. | | | | | | |
| ^c^ Includes household contribution to employer-sponsored insurance premiums, direct purchase of insurance, and medical portion of property and casualty insurance. | | | | | | |
| ^d^ Other private sector includes employers (e.g. employer sponsored health insurance, worksite health, workers' compensation), hospitals, nonprofits, health-related philanthrophic support. These data came from eTable 4, where we referred to this sector simply as "business" to match CMS naming convention. | | | | | | |
| Source: CMS. 2015 Actuarial report on the Financial outlook for medicaid. https://www.medicaid.gov/medicaid/financing-and-reimbursement/downloads/medicaid-actuarial-report-2015.pdf Carroll, Aaron. 2014. "Medicaid gives the poor a reason to say no thanks." NYTimes. https://www.nytimes.com/2014/09/23/upshot/medicaid-gives-the-poor-a-reason-to-say-no-thanks.html Center for Medicare Advocacy. "Medicare cost-sharing for dual-eligibles: who pays what for whom?" http://www.medicareadvocacy.org/medicare-cost-sharing-for-dual-eligibles-who-pays-what-for-whom/ | | | | | | |
