# Supplementary material for: Cardiometabolic disease costs associated with suboptimal diet in the United States: A cost analysis based on a microsimulation model
Source: PLoS Med. 2019 Dec 17;16(12):e1002981. doi: 10.1371/journal.pmed.1002981 (PMC6917211; doi:10.1371/journal.pmed.1002981)
Supplement: S6 Table — (DOCX) [file pmed.1002981.s015.docx]

| **S6 Table. 2014 Personal Healthcare Expenditure, by Sources of Payments and Ultimate Cost-Bearer** | | |
| --- | --- | --- |
|  |  | Expenditure (in Billions of US$) |
| Total |  | 2563.83 |
| Government | Subtotal | 1352.47 |
|  | Private insurance^a,b^ | 172.87 |
|  | Medicaid | 444.90 |
|  | Medicare^c^ | 580.89 |
|  | CHIP | 10.90 |
|  | Defense | 37.89 |
|  | VA | 57.13 |
|  | Other programs^d^ | 47.89 |
| Businesses | Subtotal | 603.89 |
|  | Private insurance^b^ | 418.27 |
|  | Worksite health & workers' compensation | 52.95 |
|  | Other private revenue^e^ | 132.67 |
| Households | Subtotal | 607.47 |
|  | Out of Pocket | 329.80 |
|  | Private insurance^b,f^ | 277.67 |
| ^a^ Includes federal government contribution to employer-sponsored insurance premiums, marketplace tax credits and subsidies, retiree drug subsidy payments to employer-sponsored health insurance plan, state & local government contribution to employer-sponsored insurance. | | |
| ^b^ Profits to insurance companies are deducted as they should not be counted toward the US Personal Health Expenditure. Because CMS does not provide information on how to accurately attribute profits by ultimate cost-bearer, we assumed that government, businesses and households bore the costs of these profits equally. | | |
| ^c^ Traditionally CMS divides Medicare expenditure by government (including federal government paid employer Medicare Hospital Insurance Trust Fund Payroll Taxes, federal general revenue and Medicare Net Trust Fund Expenditures, federal portion of medicare buy-in premiums, state employer medicare hospital insurance trust fund payroll taxes, state phase down payments, state portion of Medicare buy-in premiums), businesses (i.e. employer Medicare Hospital Insurance Trust Fund Payroll Taxes), and households (including employee and self-employment payroll taxes and voluntary premiums paid to Medicare hospital insurance trust fund and premiums paid by individuals to Medicare supplementary medical insurance trust fund.) Here we counted all Medicare expenditure as government expenses, as health care cost saving from an SSB tax will unlikely result in a reduction in Medicare payroll taxes. | | |
| ^d^ Other programs include Indian Health Services, General Assistance, Maternal and/Child Health, Vocational Rehabilitation, SAMHSA, School Health and other federal, state and local programs; | | |
| ^e^ Includes health-related philanthropic support, nonoperating revenue, investment income, and privately-funded structures and equipment; | | |
| ^f^ Includes household contribution to employer-sponsored insurance premiums, direct purchase of insurance, and medical portion of property and casualty insurance. | | |
