# Supplementary material for: Cardiometabolic disease costs associated with suboptimal diet in the United States: A cost analysis based on a microsimulation model
Source: PLoS Med. 2019 Dec 17;16(12):e1002981. doi: 10.1371/journal.pmed.1002981 (PMC6917211; doi:10.1371/journal.pmed.1002981)
Supplement: S7 Table — (DOCX) [file pmed.1002981.s016.docx]

| **S7 Table.** **Dietary factors associated with cardiometabolic outcomes and standardized magnitudes of effect sizes** | | | | | | | | | | |
| --- | --- | --- | --- | --- | --- | --- | --- | --- | --- | --- |
|  |  |  |  |  | Relative Risk by Age, years | | | | | |
|  | Units of RR | Optimal | Mean Consumption | Outcome | 25-34 | 35-44 | 45-54 | 55-64 | 65-74 | ≥75 |
| Fruits excluding  fruit juices, grams/day | 100 | 300 | 120.6 | RR CHD | 0.92 | 0.92 | 0.93 | 0.94 | 0.95 | 0.97 |
|  |  |  |  | RR Ischemic Stroke | 0.83 | 0.83 | 0.86 | 0.88 | 0.90 | 0.94 |
|  |  |  |  | RR Hemorrhagic Stroke | 0.63 | 0.64 | 0.69 | 0.73 | 0.77 | 0.86 |
| Vegetables including legumes, grams/day | 100 | 400 | 188.2 | RR CHD | 0.93 | 0.93 | 0.94 | 0.95 | 0.96 | 0.98 |
|  |  |  |  | RR Ischemic Stroke | 0.76 | 0.77 | 0.80 | 0.83 | 0.86 | 0.92 |
|  |  |  |  | RR Hemorrhagic Stroke | 0.76 | 0.77 | 0.8 | 0.83 | 0.86 | 0.92 |
| Nuts/Seeds | 1 oz/week | 20.2 grams/day 5 oz/week | 12.5 grams/day | RR Diabetes | 0.95 | 0.95 | 0.96 | 0.97 | 0.97 | 0.98 |
| Whole grains, grams/day | 50 | 125 | 21.9 | RR CHD | 0.95 | 0.95 | 0.96 | 0.97 | 0.97 | 0.98 |
|  |  |  |  | RR Ischemic Stroke | 0.88 | 0.88 | 0.90 | 0.91 | 0.93 | 0.96 |
|  |  |  |  | RR Hemorrhagic Stroke | 0.88 | 0.88 | 0.90 | 0.91 | 0.93 | 0.96 |
|  |  |  |  | RR Diabetes | 0.83 | 0.83 | 0.86 | 0.88 | 0.90 | 0.94 |
| Red meats, unprocessed, grams/day | 100 | 14.3 | 46.9 | RR Diabetes | 1.30 | 1.29 | 1.24 | 1.19 | 1.16 | 1.09 |
| Processed meats, grams/day | 50 | 0 | 30.7 | RR CHD | 1.62 | 1.58 | 1.47 | 1.38 | 1.3 | 1.16 |
|  |  |  |  | RR Diabetes | 1.86 | 1.81 | 1.65 | 1.52 | 1.41 | 1.22 |
| SSBs, 8-oz servings/day (baseline BMI <25) | 1 | 0 | 1 | kg/m2 | 0.1 | 0.1 | 0.1 | 0.1 | 0.1 | 0.1 |
| SSBs, 8-oz servings/day (baseline BMI >=25) | 1 | 0 | 1 | kg/m2 | 0.23 | 0.23 | 0.23 | 0.23 | 0.23 | 0.23 |
| SSBs, 8-oz servings/day | 1 | 0 | 1 | RR CHD | 1.33 | 1.31 | 1.26 | 1.21 | 1.17 | 1.09 |
|  |  |  |  | RR Diabetes (BMI adjusted) | 1.35 | 1.33 | 1.27 | 1.22 | 1.18 | 1.10 |
|  |  |  |  | RR Diabetes (BMI unadjusted) | 1.55 | 1.52 | 1.43 | 1.34 | 1.28 | 1.15 |
| SSBs | 5 kg/m2 increase in BMI | 0 8-oz servings/day | 1 8-oz servings/day | RR CHD | 1.45 | 1.42 | 1.35 | 1.28 | 1.23 | 1.13 |
|  |  |  |  | RR Stroke | 1.24 | 1.23 | 1.19 | 1.16 | 1.13 | 1.07 |
|  |  |  |  | RR Diabetes | 3.55 | 3.07 | 2.66 | 2.32 | 2.03 | 1.52 |
| PUFA replacing Carbs | 5 % energy/day | 11 % energy replacing SFA | 7.8 % energy replacing SFA | RR CHD | 0.86 | 0.86 | 0.88 | 0.90 | 0.92 | 0.95 |
| PUFA replacing SFA | 5 % energy/day | 11 % energy replacing SFA | 7.8 % energy replacing SFA | RR CHD | 0.87 | 0.87 | 0.89 | 0.91 | 0.92 | 0.96 |
| Seafood omega3, mgrams/day | 100 | 250 | 98.1 | RR CHD | 0.79 | 0.80 | 0.82 | 0.85 | 0.87 | 0.93 |
|  |  |  |  |  | Blood pressure changes | | | | | |
| Sodium, mgrams/day | 2300 | 2000 | 3481.5 | SBP, main effect, white normotensive (mmHg) | 1.64 | 2.69 | 3.74 | 4.79 | 5.84 | 5.84 |
|  |  |  |  | SBP blacks, additional effect (mmHg) | 2.49 | 2.49 | 2.49 | 2.49 | 2.49 | 2.49 |
|  |  |  |  | SBP HTN, additional effect (mmHg) | 1.87 | 1.87 | 1.87 | 1.87 | 1.87 | 1.87 |

Abbreviations: RR, relative risk; CHD, coronary heart disease; BMI, body mass index; SSB, sugar-sweetened beverage; PUFA, polyunsaturated fat; SFA, saturated fatty acid; SBP, systolic blood pressure; HTN, hypertension.
