# Supplementary material for: Cardiometabolic disease costs associated with suboptimal diet in the United States: A cost analysis based on a microsimulation model
Source: PLoS Med. 2019 Dec 17;16(12):e1002981. doi: 10.1371/journal.pmed.1002981 (PMC6917211; doi:10.1371/journal.pmed.1002981)
Supplement: S8 Table — (DOCX) [file pmed.1002981.s017.docx]

| **S8 Table. Five Year Health Outcomes (per million) by food/nutrient group** | | | | | | | |
| --- | --- | --- | --- | --- | --- | --- | --- |
|  |  | **Total No. of Events** | **MI Events** | **CVA Events** | **Total No. of Deaths** | **IHD Deaths** | **CVA Deaths** |
|  | **Usual** | 40,755 | **18,227** | **22,528** | 11,892 | **7,382** | **4,510** |
| Fruits excluding fruit juices, grams/day | Optimal | 33,168 | 16,143 | 17,025 | 10,926 | 6,933 | 3,993 |
|  | **Diff.** | **7,587** | **2,084** | **5,503** | **966** | **449** | **517** |
| Vegetables including legumes, grams/day | Optimal | 32,050 | 16,217 | 15,833 | 10,829 | 6,956 | 3,873 |
|  | **Diff.** | **8,705** | **2,010** | **6,695** | **1,063** | **426** | **637** |
| Nuts/seeds, grams/day | Optimal | 36,397 | 13,864 | 22,533 | 10,864 | 6,343 | 4,521 |
|  | **Diff.** | **4,358** | **4,363** | **-5** | **1,028** | **1,039** | **-11** |
| Whole grains, grams/day | Optimal | 36,083 | 16,936 | 19,147 | 11,313 | 7,120 | 4,193 |
|  | **Diff.** | **4,672** | **1,291** | **3,381** | **579** | **262** | **317** |
| Red meats, unprocessed, grams/day | Optimal | 40,727 | 18,172 | 22,555 | 11,897 | 7,383 | 4,514 |
|  | **Diff.** | **28** | **55** | **-27** | **-5** | **-1** | **-4** |
| Processed meats, grams/day | Optimal | 37,675 | 15,132 | 22,543 | 11,188 | 6,669 | 4,519 |
|  | **Diff.** | **3,080** | **3,095** | **-15** | **704** | **713** | **-9** |
| SSBs, 8-oz servings/day | Optimal | 37,403 | 15,472 | 21,931 | 11,224 | 6,758 | 4,466 |
|  | **Diff.** | **3,352** | **2,755** | **597** | **668** | **624** | **44** |
| PUFAs, % energy replacing carbohydrates or saturated fats | Optimal | 39,595 | 17,037 | 22,558 | 11,625 | 7,106 | 4,519 |
|  | **Diff.** | **1,160** | **1,190** | **-30** | **267** | **276** | **-9** |
| Seafood omega-3 fats, mgrams/day | Optimal | 36,484 | 13,934 | 22,550 | 10,855 | 6,332 | 4,523 |
|  | **Diff.** | **4,271** | **4,293** | **-22** | **1,037** | **1,050** | **-13** |
| Sodium, mgrams/day | Optimal | 39,032 | 17,601 | 21,431 | 11,454 | 7,078 | 4,376 |
|  | **Diff.** | **1,723** | **626** | **1,097** | **438** | **304** | **134** |

Abbreviations: MI, myocardial infarction; CVA, cerebrovascular; IHD, ischemic heart disease; SSB, sugar-sweetened beverage; PUFA, polyunsaturated fat.
