# Supplementary material for: Cardiometabolic disease costs associated with suboptimal diet in the United States: A cost analysis based on a microsimulation model
Source: PLoS Med. 2019 Dec 17;16(12):e1002981. doi: 10.1371/journal.pmed.1002981 (PMC6917211; doi:10.1371/journal.pmed.1002981)
Supplement: S9 Table — (DOCX) [file pmed.1002981.s018.docx]

| **S9 Table. Five Year Health Outcomes (per million) by sex, age, race and health insurance** | | | | | | | | |
| --- | --- | --- | --- | --- | --- | --- | --- | --- |
|  |  |  | **Total No. of Events** | **MI Events** | **CVA Events** | **Total No. of Deaths** | **IHD Deaths** | **CVA Deaths** |
| **Overall** | | Usual | 40,755 | 18,227 | 22,528 | 11,892 | 7,382 | 4,510 |
|  |  | Optimal | 15,709 | 5,812 | 9,897 | 7,296 | 4,080 | 3,216 |
|  |  | **Diff.** | **25,046** | **12,415** | **12,631** | **4,596** | **3,302** | **1,294** |
| **Sex** | Male | Usual | 49,137 | 24,990 | 24,147 | 15,145 | 10,356 | 4,789 |
|  |  | Optimal | 17,531 | 7,845 | 9,686 | 8,754 | 5,481 | 3,273 |
|  |  | **Diff.** | **31,606** | **17,145** | **14,461** | **6,391** | **4,875** | **1,516** |
|  | Female | Usual | 32,857 | 11,825 | 21,032 | 9,204 | 4,936 | 4,268 |
|  |  | Optimal | 13,973 | 4,138 | 9,835 | 5,841 | 2,782 | 3,059 |
|  |  | **Diff.** | **18,884** | **7,687** | **11,197** | **3,363** | **2,154** | **1,209** |
| **Age Group** | <65 years | Usual | 27,200 | 13,553 | 13,647 | 6,043 | 4,334 | 1,709 |
|  |  | Optimal | 7,563 | 3,077 | 4,486 | 2,336 | 1,501 | 835 |
|  |  | Diff. | **19,637** | **10,476** | **9,161** | **3,707** | **2,833** | **874** |
|  | ≥65 years | Usual | 95,716 | 38,600 | 57,116 | 34,344 | 20,217 | 14,127 |
|  |  | Optimal | 46,167 | 16,561 | 29,606 | 24,869 | 13,717 | 11,152 |
|  |  | **Diff.** | **49,549** | **22,039** | **27,510** | **9,475** | **6,500** | **2,975** |
| **Race^a^** | White | Usual | 41,954 | 18,647 | 23,307 | 12,797 | 8,011 | 4,786 |
|  |  | Optimal | 16,897 | 6,522 | 10,375 | 7,956 | 4,641 | 3,315 |
|  |  | **Diff.** | **25,057** | **12,125** | **12,932** | **4,841** | **3,370** | **1,471** |
|  | African Americans | Usual | 42,860 | 18,285 | 24,575 | 10,446 | 5,642 | 4,804 |
|  |  | Optimal | 13,623 | 4,481 | 9,142 | 5,818 | 2,418 | 3,400 |
|  |  | **Diff.** | **29,237** | **13,804** | **15,433** | **4,628** | **3,224** | **1,404** |
|  | Hispanic | Usual | 33,786 | 15,796 | 17,990 | 9,000 | 5,903 | 3,097 |
|  |  | Optimal | 11,730 | 4,343 | 7,387 | 4,583 | 2,616 | 1,967 |
|  |  | **Diff.** | **22,056** | **11,453** | **10,603** | **4,417** | **3,287** | **1,130** |
| **Education^b^** | <High school | Usual | **54,221** | **24,327** | **29,894** | **16,741** | **9,919** | **6,822** |
|  |  | Optimal | **19,757** | **7,351** | **12,406** | **10,780** | **5,665** | **5,115** |
|  |  | **Diff.** | **34,464** | **16,976** | **17,488** | **5,961** | **4,254** | **1,707** |
|  | High school | Usual | **42,895** | **19,572** | **23,323** | **12,682** | **8,095** | **4,587** |
|  |  | Optimal | **16,023** | **6,146** | **9,877** | **7,391** | **4,265** | **3,126** |
|  |  | **Diff.** | **26,872** | **13,426** | **13,446** | **5,291** | **3,830** | **1,461** |
|  | College | Usual | **29,947** | **12,983** | **16,964** | **8,396** | **5,291** | **3,105** |
|  |  | Optimal | **12,565** | **4,840** | **7,725** | **5,010** | **2,934** | **2,076** |
|  |  | **Diff.** | **17,383** | **8,143** | **9,239** | **3,386** | **2,357** | **1,029** |
| **Health Insurance^c^** | Private | Usual | 26,618 | 12,221 | 14,397 | 6,920 | 4,771 | 2,149 |
|  |  | Optimal | 8,911 | 3,408 | 5,503 | 3,140 | 2,005 | 1,135 |
|  |  | **Diff.** | **17,707** | **8,813** | **8,894** | **3,780** | **2,766** | **1,014** |
|  | Medicare | Usual | 85,222 | 34,321 | 50,901 | 31,035 | 17,539 | 13,496 |
|  |  | Optimal | 41,067 | 14,702 | 26,365 | 22,982 | 12,268 | 10,714 |
|  |  | **Diff.** | **44,155** | **19,619** | **24,536** | **8,053** | **5,271** | **2,782** |
|  | Medicaid | Usual | 38,014 | 17,983 | 20,031 | 8,921 | 5,004 | 3,917 |
|  |  | Optimal | 10,666 | 4,116 | 6,550 | 4,785 | 1,923 | 2,862 |
|  |  | **Diff.** | **27,348** | **13,867** | **13,481** | **4,136** | **3,081** | **1,055** |
|  | Dual Eligible | Usual | 87,668 | 41,115 | 46,553 | 23,500 | 13,337 | 10,163 |
|  |  | Optimal | 33,580 | 14,316 | 19,264 | 17,671 | 9,417 | 8,254 |
|  |  | **Diff.** | **54,088** | **26,799** | **27,289** | **5,829** | **3,920** | **1,909** |
|  | Other Government | Usual | 37,692 | 17,585 | 20,107 | 8,990 | 5,728 | 3,262 |
|  |  | Optimal | 12,848 | 5,482 | 7,366 | 4,911 | 2,797 | 2,114 |
|  |  | **Diff.** | **24,844** | **12,103** | **12,741** | **4,079** | **2,931** | **1,148** |
|  | No Coverage | Usual | 33,331 | 17,975 | 15,356 | 7,488 | 5,610 | 1,878 |
|  |  | Optimal | 8,500 | 3,587 | 4,913 | 2,756 | 1,961 | 795 |
|  |  | **Diff.** | **24,831** | **14,388** | **10,443** | **4,732** | **3,649** | **1,083** |

Abbreviations: MI, myocardial infarction; CVA, cerebrovascular; IHD, ischemic heart disease.

^a^Race - White: non‐Hispanic White; Black: non‐Hispanic Black; Hispanic: Mexican American/other Hispanic.

^b^Education - <High‐school: less than high school degree; High‐school: high school degree/equivalent or some college; College: ≥4‐year college degree.

^c^Health insurance - Private includes: private, single service plan, private plus other government, other coverage; Medicare includes: Medicare, Medi-Gap, Medicare plus other government, Medicare plus private; Medicaid includes only Medicaid; Dual eligible includes: Medicare plus Medicaid; and Other government includes: other government; state-sponsored; military.
