# Supplementary material for: Cardiometabolic disease costs associated with suboptimal diet in the United States: A cost analysis based on a microsimulation model
Source: PLoS Med. 2019 Dec 17;16(12):e1002981. doi: 10.1371/journal.pmed.1002981 (PMC6917211; doi:10.1371/journal.pmed.1002981)
Supplement: S10 Table — (DOCX) [file pmed.1002981.s019.docx]

| **S10 Table. One Year Health Outcomes (per million) by food/nutrient group** | | | | | | | |
| --- | --- | --- | --- | --- | --- | --- | --- |
|  |  | **Total No. of Events** | **MI Events** | **CVA Events** | **Total No. of Deaths** | **IHD Deaths** | **CVA Deaths** |
|  | **Usual** | **8,151** | **3,645** | **4,506** | **2,378** | **1,476** | **902** |
| Fruits excluding fruit juices, grams/day | Optimal | 6,634 | 3,229 | 3,405 | 2,185 | 1,387 | 799 |
|  | **Diff.** | **1,517** | **417** | **1,101** | **193** | **90** | **103** |
| Vegetables including legumes, grams/day | Optimal | 6,410 | 3,243 | 3,167 | 2,166 | 1,391 | 775 |
|  | **Diff.** | **1,741** | **402** | **1,339** | **213** | **85** | **127** |
| Nuts/seeds, grams/day | Optimal | 7,279 | 2,773 | 4,507 | 2,173 | 1,269 | 904 |
|  | **Diff.** | **872** | **873** | **-1** | **206** | **208** | **-2** |
| Whole grains, grams/day | Optimal | 7,217 | 3,387 | 3,829 | 2,263 | 1,424 | 839 |
|  | **Diff.** | **934** | **258** | **676** | **116** | **52** | **63** |
| Red meats, unprocessed, grams/day | Optimal | 8,145 | 3,634 | 4,511 | 2,379 | 1,477 | 903 |
|  | **Diff.** | **6** | **11** | **-5** | **-1** | **0** | **-1** |
| Processed meats, grams/day | Optimal | 7,535 | 3,026 | 4,509 | 2,238 | 1,334 | 904 |
|  | **Diff.** | **616** | **619** | **-3** | **141** | **143** | **-2** |
| SSBs, 8-oz servings/day | Optimal | 7,481 | 3,094 | 4,386 | 2,245 | 1,352 | 893 |
|  | **Diff.** | **670** | **551** | **119** | **134** | **125** | **9** |
| PUFAs, % energy replacing carbohydrates or saturated fats | Optimal | 7,919 | 3,407 | 4,512 | 2,325 | 1,421 | 904 |
|  | **Diff.** | **232** | **238** | **-6** | **53** | **55** | **-2** |
| Seafood omega-3 fats, mgrams/day | Optimal | 7,297 | 2,787 | 4,510 | 2,171 | 1,266 | 905 |
|  | **Diff.** | **854** | **859** | **-4** | **207** | **210** | **-3** |
| Sodium, mgrams/day | Optimal | 7,806 | 3,520 | 4,286 | 2,291 | 1,416 | 875 |
|  | **Diff.** | **345** | **125** | **219** | **88** | **61** | **27** |

Abbreviations: MI, myocardial infarction; CVA, cerebrovascular; IHD, ischemic heart disease; SSB, sugar-sweetened beverage; PUFA, polyunsaturated fat.
