# Supplementary material for: Cardiometabolic disease costs associated with suboptimal diet in the United States: A cost analysis based on a microsimulation model
Source: PLoS Med. 2019 Dec 17;16(12):e1002981. doi: 10.1371/journal.pmed.1002981 (PMC6917211; doi:10.1371/journal.pmed.1002981)
Supplement: S11 Table — (DOCX) [file pmed.1002981.s020.docx]

| **S11 Table. One Year Health Outcomes (per million)** **by sex, age group, race and health insurance** | | | | | | | | |
| --- | --- | --- | --- | --- | --- | --- | --- | --- |
|  |  |  | **Total No. of Events** | **MI Events** | **CVA Events** | **Total No. of Deaths** | **IHD Deaths** | **CVA Deaths** |
| **Overall** | | Usual | 8,151 | 3,645 | 4,506 | 2,378 | 1,476 | 902 |
|  |  | Optimal | 3,142 | 1,162 | 1,979 | 1,459 | 816 | 643 |
|  |  | **Diff.** | **5,009** | **2,483** | **2,526** | **919** | **660** | **259** |
| **Sex** | Male | Usual | 9,827 | 4,998 | 4,829 | 3,029 | 2,071 | 958 |
|  |  | Optimal | 3,506 | 1,569 | 1,937 | 1,751 | 1,096 | 655 |
|  |  | **Diff.** | **6,321** | **3,429** | **2,892** | **1,278** | **975** | **303** |
|  | Female | Usual | 6,571 | 2,365 | 4,206 | 1,841 | 987 | 854 |
|  |  | Optimal | 2,795 | 828 | 1,967 | 1,168 | 556 | 612 |
|  |  | **Diff.** | **3,777** | **1,537** | **2,239** | **673** | **431** | **242** |
| **Age Group** | <65 years | Usual | 5,440 | 2,711 | 2,729 | 1,209 | 867 | 342 |
|  |  | Optimal | 1,513 | 615 | 897 | 467 | 300 | 167 |
|  |  | Diff. | 3,927 | 2,095 | 1,832 | 741 | 567 | 175 |
|  | ≥65 years | Usual | 19,143 | 7,720 | 11,423 | 6,869 | 4,043 | 2,825 |
|  |  | Optimal | 9,233 | 3,312 | 5,921 | 4,974 | 2,743 | 2,230 |
|  |  | **Diff.** | **9,910** | **4,408** | **5,502** | **1,895** | **1,300** | **595** |
| **Race^a^** | White | Usual | 8,391 | 3,729 | 4,661 | 2,559 | 1,602 | 957 |
|  |  | Optimal | 3,379 | 1,304 | 2,075 | 1,591 | 928 | 663 |
|  |  | **Diff.** | **5,011** | **2,425** | **2,586** | **968** | **674** | **294** |
|  | African Americans | Usual | 8,572 | 3,657 | 4,915 | 2,089 | 1,128 | 961 |
|  |  | Optimal | 2,725 | 896 | 1,828 | 1,164 | 484 | 680 |
|  |  | **Diff.** | **5,847** | **2,761** | **3,087** | **926** | **645** | **281** |
|  | Hispanic | Usual | 6,757 | 3,159 | 3,598 | 1,800 | 1,181 | 619 |
|  |  | Optimal | 2,346 | 869 | 1,477 | 917 | 523 | 393 |
|  |  | **Diff.** | **4,411** | **2,291** | **2,121** | **883** | **657** | **226** |
| **Education^b^** | <High school | Usual | **1844** | **4865** | **5979** | **3348** | **1984** | **1364** |
|  |  | Optimal | **3951** | **1470** | **2481** | **2156** | **1133** | **1023** |
|  |  | **Diff.** | **6893** | **3395** | **3498** | **1192** | **851** | **341** |
|  | High school | Usual | **8579** | **3914** | **4665** | **2536** | **1619** | **917** |
|  |  | Optimal | **3205** | **1229** | **1975** | **1478** | **853** | **625** |
|  |  | **Diff.** | **5374** | **2685** | **2689** | **1058** | **766** | **292** |
|  | College | Usual | **5989** | **2597** | **3393** | **1679** | **1058** | **621** |
|  |  | Optimal | **2513** | **968** | **1545** | **1002** | **587** | **415** |
|  |  | **Diff.** | **3476** | **1629** | **1848** | **677** | **471** | **206** |
| **Health Insurance^c^** | Private | Usual | 5,324 | 2,444 | 2,879 | 1,384 | 954 | 430 |
|  |  | Optimal | 1,782 | 682 | 1,101 | 628 | 401 | 227 |
|  |  | **Diff.** | **3,541** | **1,763** | **1,779** | **756** | **553** | **203** |
|  | Medicare | Usual | 17,044 | 6,864 | 10,180 | 6,207 | 3,508 | 2,699 |
|  |  | Optimal | 8,213 | 2,940 | 5,273 | 4,596 | 2,454 | 2,143 |
|  |  | **Diff.** | **8,831** | **3,924** | **4,907** | **1,611** | **1,054** | **556** |
|  | Medicaid | Usual | 7,603 | 3,597 | 4,006 | 1,784 | 1,001 | 783 |
|  |  | Optimal | 2,133 | 823 | 1,310 | 957 | 385 | 572 |
|  |  | **Diff.** | **5,470** | **2,773** | **2,696** | **827** | **616** | **211** |
|  | Dual Eligible | Usual | 17,534 | 8,223 | 9,311 | 4,700 | 2,667 | 2,033 |
|  |  | Optimal | 6,716 | 2,863 | 3,853 | 3,534 | 1,883 | 1,651 |
|  |  | **Diff.** | **10,818** | **5,360** | **5,458** | **1,166** | **784** | **382** |
|  | Other Government | Usual | 7,538 | 3,517 | 4,021 | 1,798 | 1,146 | 652 |
|  |  | Optimal | 2,570 | 1,096 | 1,473 | 982 | 559 | 423 |
|  |  | **Diff.** | **4,969** | **2,421** | **2,548** | **816** | **586** | **230** |
|  | No Coverage | Usual | 6,666 | 3,595 | 3,071 | 1,498 | 1,122 | 376 |
|  |  | Optimal | 1,700 | 717 | 983 | 551 | 392 | 159 |
|  |  | **Diff.** | **4,966** | **2,878** | **2,089** | **946** | **730** | **217** |

Abbreviations: MI, myocardial infarction; CVA, cerebrovascular; IHD, ischemic heart disease.

^a^Race - White: non‐Hispanic White; Black: non‐Hispanic Black; Hispanic: Mexican American/other Hispanic.

^b^Education - <High‐school: less than high school degree; High‐school: high school degree/equivalent or some college; College: ≥4‐year college degree.

^c^Health insurance - Private includes: private, single service plan, private plus other government, other coverage; Medicare includes: Medicare, Medi-Gap, Medicare plus other government, Medicare plus private; Medicaid includes only Medicaid; Dual eligible includes: Medicare plus Medicaid; and Other government includes: other government; state-sponsored; military.
