# Supplementary material for: Cardiometabolic disease costs associated with suboptimal diet in the United States: A cost analysis based on a microsimulation model
Source: PLoS Med. 2019 Dec 17;16(12):e1002981. doi: 10.1371/journal.pmed.1002981 (PMC6917211; doi:10.1371/journal.pmed.1002981)
Supplement: S12 Table — (DOCX) [file pmed.1002981.s021.docx]

| **S12 Table.** **Five years dietary related costs of cardiometabolic disease per adults (US$) by food/nutrient group** | | | | | |
| --- | --- | --- | --- | --- | --- |
|  |  | **Total Cost** | **Acute^a^** | **Chronic^b^** | **Drug^c^** |
|  | **Usual** | **6,437** | **1,807** | **4,054** | **577** |
| Overall | Optimal | 5,037 | 629 | 3,853 | 555 |
|  | **Diff.** | **1,400** | **1,178** | **201** | **22** |
| Fruits excluding fruit juices, grams/day | Optimal | 6,170 | 1,561 | 4,034 | 575 |
|  | **Diff.** | **267** | **245** | **20** | **2** |
| Vegetables including legumes, grams/day | Optimal | 6,156 | 1,549 | 4,032 | 575 |
|  | **Diff.** | **281** | **258** | **22** | **2** |
| Nuts/seeds, grams/day | Optimal | 6,065 | 1,480 | 4,009 | 576 |
|  | **Diff.** | **373** | **326** | **45** | **1** |
| Whole grains, grams/day | Optimal | 6,228 | 1,657 | 3,996 | 576 |
|  | **Diff.** | **209** | **150** | **58** | **1** |
| Red meats, unprocessed, grams/day | Optimal | 6,423 | 1,805 | 4,041 | 577 |
|  | **Diff.** | **15** | **2** | **12** | **0** |
| Processed meats, grams/day | Optimal | 6,153 | 1,577 | 4,000 | 576 |
|  | **Diff.** | **285** | **229** | **54** | **1** |
| SSBs, 8-oz servings/day | Optimal | 6168 | 1589 | 4003 | 576 |
|  | **Diff.** | **270** | **218** | **51** | **1** |
| PUFAs,% energy replacing carbohydrates or saturated fats | Optimal | 6343 | 1719 | 4047 | 576 |
|  | **Diff.** | **95** | **88** | **6** | **0** |
| Seafood omega-3 fats, mgrams/day | Optimal | 6083 | 1478 | 4029 | 576 |
|  | **Diff.** | **355** | **329** | **25** | **1** |
| Sodium, mgrams/day | Optimal | 6330 | 1725 | 4046 | 560 |
|  | **Diff.** | **107** | **82** | **8** | **17** |

Abbreviations: SSB, sugar-sweetened beverage; PUFA, polyunsaturated fat.

^a^Acute costs: related to the acute hospitalization event.

^b^Chronic costs: not related to the acute event or drugs.

^c^Drug costs: drug-related costs.
