# Supplementary material for: Cardiometabolic disease costs associated with suboptimal diet in the United States: A cost analysis based on a microsimulation model
Source: PLoS Med. 2019 Dec 17;16(12):e1002981. doi: 10.1371/journal.pmed.1002981 (PMC6917211; doi:10.1371/journal.pmed.1002981)
Supplement: S13 Table — (DOCX) [file pmed.1002981.s022.docx]

| **S13 Table. Five years related costs of cardiometabolic disease per adults (US$) by sex, age group, race, BMI, and health insurance** | | | | | | |
| --- | --- | --- | --- | --- | --- | --- |
|  |  |  | **Total Cost** | **Acute^a^** | **Chronic^b^** | **Drug^c^** |
| **Sex** | Male | Usual | 7,885 | 2,409 | 4,789 | 688 |
|  |  | Optimal | 5,980 | 792 | 4,528 | 660 |
|  |  | **Diff.** | **1,905** | **1,616** | **260** | **28** |
|  | Female | Usual | 6,008 | 1,494 | 3,957 | 558 |
|  |  | Optimal | 4,903 | 576 | 3,787 | 539 |
|  |  | **Diff.** | **1,105** | **918** | **169** | **18** |
| **Age Group** | 35-49 years | Usual | 2,808 | 646 | 1,787 | 377 |
|  |  | Optimal | 2,088 | 118 | 1,606 | 364 |
|  |  | **Diff.** | **721** | **527** | **181** | **13** |
|  | 50-64 years | Usual | 6,723 | 1,920 | 4,235 | 568 |
|  |  | Optimal | 5,098 | 532 | 4,026 | 541 |
|  |  | **Diff.** | **1,625** | **1,389** | **210** | **27** |
|  | 65-80 years | Usual | 11,367 | 3,344 | 7,117 | 906 |
|  |  | Optimal | 9,331 | 1,517 | 6,935 | 879 |
|  |  | **Diff.** | **2,038** | **1,828** | **182** | **27** |
| **Race^d^** | White | Usual | 6,584 | 2,005 | 3,949 | 629 |
|  |  | Optimal | 5,086 | 746 | 3,734 | 606 |
|  |  | **Diff.** | **1,498** | **1,260** | **216** | **23** |
|  | Black | Usual | 8,716 | 1,889 | 6,162 | 665 |
|  |  | Optimal | 7,117 | 536 | 5,946 | 635 |
|  |  | **Diff.** | **1,598** | **1,352** | **216** | **30** |
|  | Hispanic | Usual | 7,180 | 1,652 | 4,972 | 555 |
|  |  | Optimal | 5,802 | 507 | 4,763 | 532 |
|  |  | **Diff.** | **1,379** | **1,146** | **209** | **24** |
|  | Others | Usual | 7,090 | 1,742 | 4,779 | 569 |
|  |  | Optimal | 5,680 | 556 | 4,571 | 552 |
|  |  | **Diff.** | **1,410** | **1,185** | **209** | **16** |
| **BMI** | <30 | Usual | 5,231 | 1,730 | 2,908 | 593 |
|  |  | Optimal | 3,928 | 654 | 2,703 | 570 |
|  |  | **Diff.** | **1,303** | **1,074** | **207** | **22** |
|  | ≥30 | Usual | 9,732 | 2,302 | 6,766 | 665 |
|  |  | Optimal | 7,930 | 749 | 6,540 | 640 |
|  |  | **Diff.** | **1,803** | **1,552** | **226** | **25** |
| **Education^e^** | <High school | Usual | 9,967 | 2,535 | 6,712 | 720 |
|  |  | Optimal | 8,008 | 834 | 6,479 | 695 |
|  |  | **Diff.** | **1,959** | **1,701** | **234** | **25** |
|  | High school | Usual | 7,270 | 2,088 | 4,537 | 645 |
|  |  | Optimal | 5,638 | 714 | 4,305 | 619 |
|  |  | **Diff.** | **1,632** | **1,375** | **231** | **26** |
|  | College | Usual | 4,658 | 1,375 | 2,761 | 524 |
|  |  | Optimal | 3,623 | 536 | 2,581 | 506 |
|  |  | **Diff.** | **1,037** | **838** | **180** | **18** |
| **Health Insurance^f^** | Private | Usual | 4,788 | 1,342 | 2,945 | 501 |
|  |  | Optimal | 3,635 | 413 | 2,743 | 479 |
|  |  | **Diff.** | **1,153** | **929** | **203** | **22** |
|  | Medicare | Usual | 13,325 | 3,950 | 8,336 | 1,038 |
|  |  | Optimal | 10,916 | 1,798 | 8,109 | 1,009 |
|  |  | **Diff.** | **2,409** | **2,153** | **227** | **29** |
|  | Medicaid | Usual | 8,684 | 1,828 | 6,281 | 576 |
|  |  | Optimal | 7,100 | 479 | 6,072 | 550 |
|  |  | **Diff.** | **1,583** | **1,349** | **209** | **26** |
|  | Dual Eligible | Usual | 17,690 | 3,814 | 12,889 | 987 |
|  |  | Optimal | 15,008 | 1,384 | 12,664 | 959 |
|  |  | **Diff.** | **2,682** | **2,429** | **225** | **28** |
|  | Other Government | Usual | 7,367 | 1,830 | 4,905 | 633 |
|  |  | Optimal | 5,864 | 566 | 4,690 | 609 |
|  |  | **Diff.** | **1,503** | **1,264** | **215** | **24** |
|  | No Coverage | Usual | 5,813 | 1,712 | 3,556 | 545 |
|  |  | Optimal | 4,230 | 395 | 3,312 | 523 |
|  |  | **Diff.** | **1,583** | **1,317** | **244** | **23** |

^a^Acute costs: related to the acute hospitalization event.

^b^Chronic costs: not related to the acute event or drugs.

^c^Drug costs: drug-related costs.

^d^Race - White: non‐Hispanic White; Black: non‐Hispanic Black; Hispanic: Mexican American/other Hispanic.

^e^Education - <High‐school: less than high school degree; High‐school: high school degree/equivalent or some college; College: ≥4‐year college degree.

^f^Health insurance - Private includes: private, single service plan, private plus other government, other coverage; Medicare includes: Medicare, Medi-Gap, Medicare plus other government, Medicare plus private; Medicaid includes only Medicaid; Dual eligible includes: Medicare plus Medicaid; and Other government includes: other government; state-sponsored; military.
